# Acoustic Communication at the Water's Edge: Evolutionary Insights from a Mudskipper

**DOI:** 10.1371/journal.pone.0021434

**Published:** 2011-06-28

**Authors:** Gianluca Polgar, Stefano Malavasi, Giacomo Cipolato, Vyron Georgalas, Jennifer A. Clack, Patrizia Torricelli

**Affiliations:** 1 Institute of Biological Sciences, Institute of Ocean and Earth Sciences, University of Malaya, Kuala Lumpur, Malaysia; 2 Department of Environmental Sciences, Informatics and Statistics, Università “Ca' Foscari” Venezia, Campo della Celestia, Venice, Italy; 3 University Museum of Zoology, Cambridge, United Kingdom; University of Western Ontario, Canada

## Abstract

Coupled behavioural observations and acoustical recordings of aggressive dyadic contests showed that the mudskipper *Periophthalmodon septemradiatus* communicates acoustically while out of water. An analysis of intraspecific variability showed that specific acoustic components may act as tags for individual recognition, further supporting the sounds' communicative value. A correlative analysis amongst acoustical properties and video-acoustical recordings in slow-motion supported first hypotheses on the emission mechanism. Acoustic transmission through the wet exposed substrate was also discussed. These observations were used to support an “exaptation hypothesis”, i.e. the maintenance of key adaptations during the first stages of water-to-land vertebrate eco-evolutionary transitions (based on eco-evolutionary and palaeontological considerations), through a comparative bioacoustic analysis of aquatic and semiterrestrial gobiid taxa. In fact, a remarkable similarity was found between mudskipper vocalisations and those emitted by gobioids and other soniferous benthonic fishes.

## Introduction

The adaptive gap between aquatic and terrestrial acoustic communication is mirrored by a lack of understanding of the eco-evolutionary mechanisms which allowed the vertebrates to cross the water-to-land ecological barrier.

Some insights come from recent paleontological studies of Devonian prototetrapods, the tetrapods' most recent common ancestors, e.g. [1], [2]. The fossil record indicated that several adaptive radiations occurred in shallow aquatic intertidal habitats of tropical deltas and flooding plains, and that key adaptations to the terrestrial environment such as limbs were apparently exaptations selected in aquatic conditions. In particular, it might be expected that during the first phases of this transition, exaptations also facilitated both the exploration of the terrestrial acoustic world, and terrestrial acoustic communication. In this respect, the Devonian tetrapod *Ichthyostega* was discovered to have a uniquely modified ear region, interpreted as an underwater acoustic receiver [3]. By contrast with the apparently aquatically adapted ear, *Ichthyostega* shows what appear to be terrestrial adaptations of the axial skeleton [4], leaving unanswered the question whether the receiver was functional also out of water. Up to date, acoustic communication was not demonstrated in Devonian tetrapods.

Other insights on the eco-evolutionary mechanisms at work along the water's edge for aquatic vertebrates may come from living species which specifically adapted to similar semi-aquatic or semi-terrestrial conditions. In particular, comparative analyses can be conducted to test an “exaptation hypothesis”, i.e. the maintenance of key acoustic adaptations during water-to-land vertebrate eco-evolutionary transitions.

Mudskippers (Teleostei: Gobiidae, or Gobionellidae *sensu* [5]: Oxudercinae) are semi-terrestrial gobies living in intertidal tropical and subtropical habitats (mangrove forests, tidal mudflats and freshwater swamps, e.g. [6], [7]), which are “fully terrestrial for some portion of their daily cycle” [8]. The habitats of these fishes are typically characterised by soft, anoxic sediments (mud to sand-mud), in which they dig their reproductive burrows, e.g. [9]. Several authors hypothesised that oxudercine gobies and Devonian prototetrapods independently evolved convergent ecological and morphological adaptations, e.g. [10], [11]. Several aspects of the palaeosynecology of Devonian prototetrapods were also considered as convergent to oxudercines' [6], [7]. Key stages of the Devonian vertebrate transition from water to land apparently occurred along the continental margins of Laurussia, in shallow aquatic intertidal habitats of tropical deltas and flooding plains, that were recently colonised by the first terrestrial plants [12]–[20]. Therefore, the habitats of these extinct forms were ecologically very similar to those occupied by mudskippers, and reasonably exerted similar selective pressures. For these reasons, mudskippers are excellent models to test the proposed guiding hypothesis.

The present phylogeny consensus includes mudskipper genera in a monophyletic clade (Oxudercinae: Periophthalmini; [8]); in fact, molecular analyses suggested that oxudercines may not constitute a monophyletic group, with some members being closer to amblyopine gobies (Amblyopinae; [5], [21]), furthermore, as in most gobioid groups, phylogenetic relationships below the genus level are presently unresolved. Nonetheless, the close relationship of oxudercines with other aquatic gobioids is supported both by morphological [8], [22] and molecular data [5], [21], [23], [24], allowing evolutionary comparative analyses at suprageneric level.

Several studies on the social behaviour and communication of *Periophthalmus* and *Boleophthalmus* spp. showed that these species are highly territorial, and make use of intense visual displays both during agonistic and reproductive intraspecific interactions, e.g. [25]–[34]. Nursall [35], [36] also investigated interspecific interactions among *Periophthalmus* spp., with emphasis on fin signalling.

The presence of acoustic communication was documented in several basal and derived aquatic gobioids, both during reproductive and aggressive encounters, e.g. [37]–[39]. Nonetheless, except for few anecdotal accounts of audible sounds produced during feeding, e.g. [40], and few behavioural and physiological reports of their hearing capacities, e.g. [41], [42], terrestrial acoustic communication has not been previously demonstrated in mudskippers.

**Figure 1:**
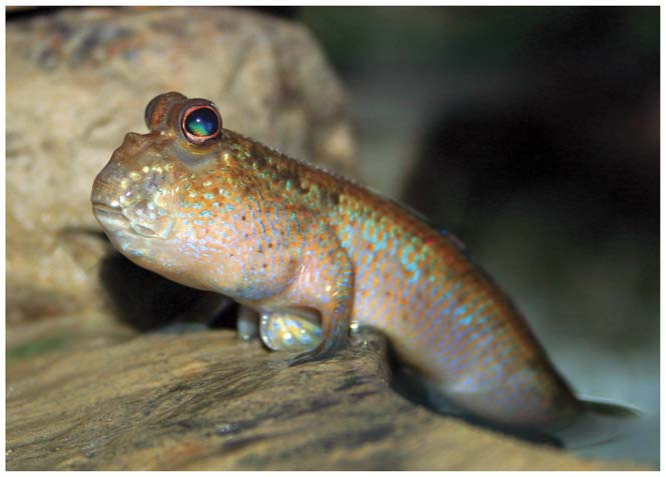
One of the males of *Periophthalmodon septemradiatus* (photo by G. Polgar).

Appropriate laboratory conditions and equipment allowed to record and analyse vocalizations of the mudskipper *Periophthalmodon septemradiatus* (Hamilton) (**Fig. 1**) during agonistic interactions, demonstrating that sounds are effectively transmitted at short distances through the wet exposed substrate. Our study aimed at: (*i*) description of the structure of the mudskipper call, assessing the main acoustical properties of the vocalisations transmitted through the prevalent transmitting medium; (*ii*) assessment of intraspecific variability in mudskipper call structure, testing also for correlation amongst acoustical properties and describing their association with visible movements (*iii*) exploring affinities of mudskipper acoustical signals with respect to other soniferous fishes, with further discussion of the possible evolutionary insights concerning the eco-evolutionary transition from aquatic to terrestrial habitats (exaptation hypothesis).

## Results

**Figure 2:**
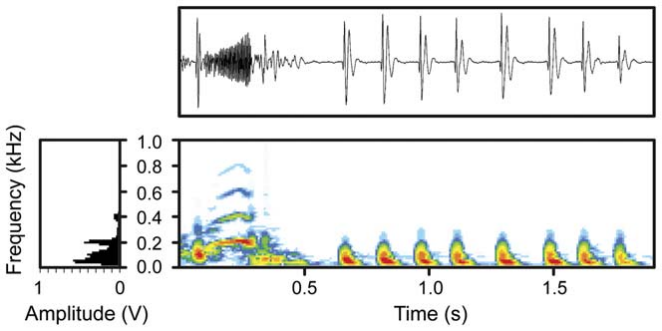
Oscillogram (top panel), spectrogram (bottom panel) and power spectrum (bottom left panel) of a representative mudskipper acoustical bout, composed of a tonal segment and nine pulsatile units; amplitude on a linear scale of 100 mV per division (arbitrary units).

**Figure 3:**
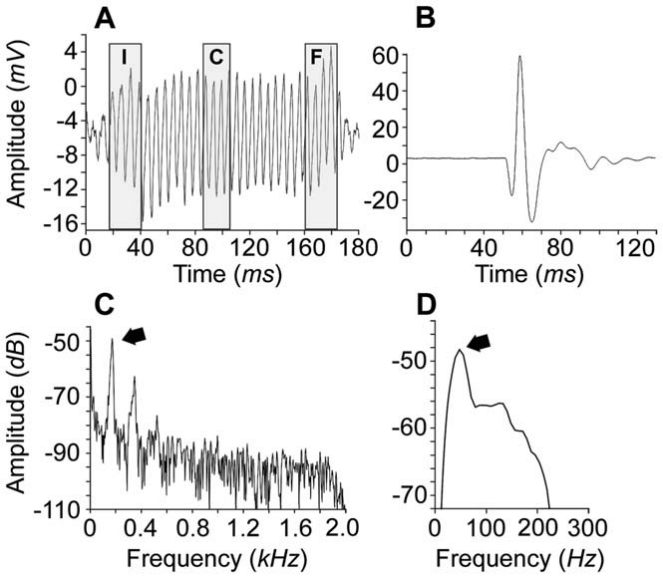
Oscillograms and spectral properties of the mudskipper calls. a, Oscillogram of a tonal segment and the three sampled portions (I: initial, C: central, F: final; each corresponding to 4 cycles) used to calculate the frequency modulation. b, Oscillogram of a pulsatile unit. c and d, power spectra of a representative tonal segment (arrow: fundamental frequency: 162 Hz) and a pulse (arrow: peak frequency: 46 Hz), respectively. Amplitude measurements: *mV*, *dB* (relative units).

**Figure 4:**
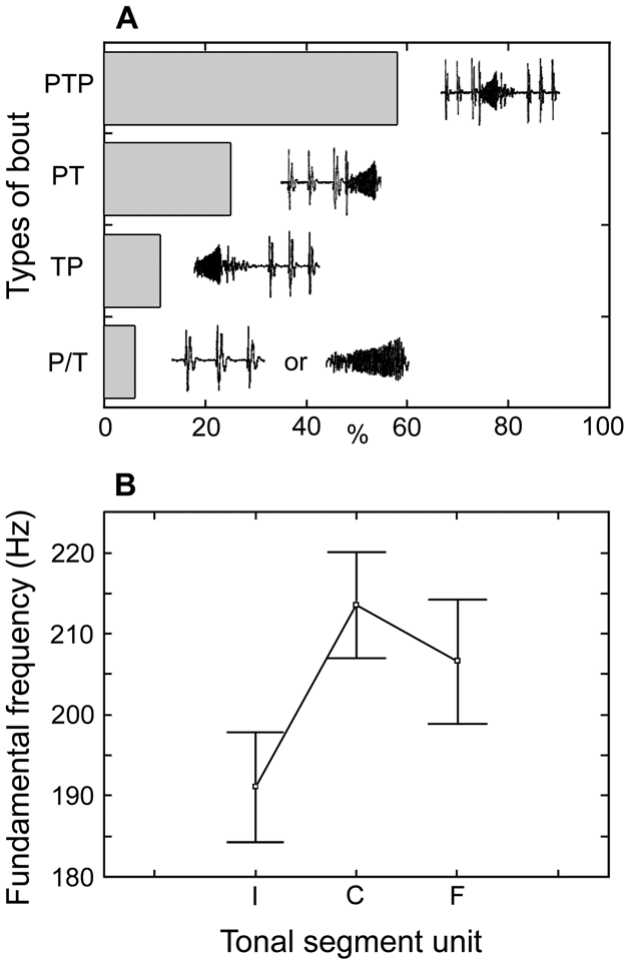
Vocal repertoire and frequency modulation. a, Repertoire and proportions of different types of bouts. PTP: tonal segments both preceded and followed by trains of pulses; PT: tonal segments preceded by trains of pulses; TP: tonal segments followed by trains of pulses; P/T: isolated pulse trains or tonal segments. b, Modulation of the fundamental frequency of tonal segments (n = 10, means ± s.e.); mean individual values were obtained from the means of each bout. Frequencies of the three portions (see **Fig. 3a** for abbreviations) were significantly different (Friedman ANOVA by ranks, df = 2, Chi Square = 10.40, p<0.01). Frequency of C and F significantly differed from I (Wilcoxon Matched Pairs test, p<0.05), but not one from each other (p = 0.20).

Vocalizations were analysed and organised in bouts, each containing different combinations of pulses and tonal segments (**Figs. 2**, **3**, **4a**; **Tables S1**, **S2**; **Fig. S1**). A small proportion of bouts contained either only trains of pulses or only tonal segments, while the majority contained both (**Fig. 4a**). Each pulse was broad band, composed by 1–3 rapidly damped cycles (**Fig. 3b**), and repeated at a relatively low rate; most pulse energy was concentrated below 100 Hz (grand mean of the peak fundamental frequency, **Table 1**, **Fig. 3d**). Tonal segments were continuous sine waves made of rapidly repeated pulses (**Fig. 3a, c**), and composed by a stronger first harmonic band (grand mean of the fundamental frequency: 168 Hz, **Table 1**) and 1–3 much weaker ones (**Fig. 2**). They were both amplitude (**Fig. 2**, top panel) and frequency modulated (**Fig. 3c**, **4b**). Within bouts, units were spaced by highly variable time intervals, although pulse-tonal intervals were much shorter than tonal-pulse intervals (**Table 1**). On average, tonal fundamental frequency, tonal frequency modulation (F-I), pulse-pulse intervals and tonal-tonal intervals were more variable within bouts than within individuals (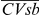
>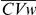; **Table 1**); all other properties showed an inverse pattern. Both within-bout and within-individual variations in duration of the single units (i.e. pulses and tonal segments) were higher than in frequency (**Fig. S2**; **Table 1**).

**Table 1:** Acoustic properties of bouts and units: grand means and coefficients of variation (CVs).

Acoustic property	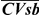	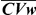	*Grand mean ± SD*	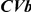	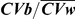
Bout duration (s)	-	80.8 (24.0–135.8)	3.2±1.6 (1.2–5.2)	50.1	0.6
Number of pulses per bout	-	85.9 (52.2–129.9)	4.8±2.1 (1.2–7.1)	42.6	0.5
Number of tonal segments per bout	-	55.5 (35.1–100.7)	1.6±0.5 (1.2–2.5)	29.1	0.5
Pulse rate (1/s)	-	55.7 (37.7–98.6)	1.8±0.6 (1.0–2.8)	32.0	0.6
Tonal rate (1/s)	-	74.4 (31.7–112.7)	1.1±0.6 (0.3–1.9)	54.4	0.7
Pulse duration (ms)	32.4 (0.0–102.2)	33.9 (18.0–85.4)	58±16 (33–76)	27.3	0.8
Pulse peak frequency (Hz)	17.8 (0.0–63.9)	20.0 (6.9–28.2)	60±6 (51–72)	10.1	0.5
Tonal duration (ms)	37.4 (6.7–88.2)	43.5 (27.8–62.1)	461±120 (279–713)	26.0	0.6
Tonal fundamental frequency (Hz)	8.3 (0.1–18.6)	6.6 (1.6–13.9)	168±14 (147–192)	8.4	1.3
Tonal fundamental frequency I	15.4 (0.3–67.4)	14.7 (9.1–36.9)	194±23 (155–229)	11.7	0.8
Tonal fundamental frequency C	13.0 (1.0–45.6)	12.3 (1.7–23.3)	219±18 (199–254)	8.3	0.7
Tonal fundamental frequency F	13.0 (0.0–47.3)	13.6 (2.1–23.4)	210±24 (182–257)	11.6	0.9
Tonal frequency modulation (C-I)	58.4 (2.1–136.0)	60.1 (30.2–82.2)	29±16 (13–62)	53.9	0.9
Tonal frequency modulation (F-C)	84.8 (20.7–149.2)	85.5 (38.6–134.4)	23±9 (9–36)	37.6	0.4
Tonal frequency modulation (F-I)	81.2 (2.7–139.5)	77.2 (59.9–105.6)	31±19 (10–70)	62.3	0.8
Pulse-pulse interval (ms)	91.3 (11.5–199.5)	76.3 (43.2–109.9)	608±384 (184–1,341)	63.1	0.8
Pulse-tonal interval (ms)	142.1 (32.6–200.0)	178.7 (88.4–300.0)	56±66 (0–181)	118.0	0.7
Tonal-pulse interval (ms)	60.7 (3.2–149.4)	61.5 (15.9–141.4)	406±202 (147–794)	49.9	0.8
Tonal-tonal interval (ms)	82.8 (12.8–137.5)	73.8 (16.2–137.1)	1000±493 (501–2,217)	49.3	0.7

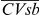
: mean within-bouts coefficient of variation ( = mean of the 78 bouts' CVs; **Fig. S2a**); 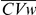: mean within-individual coefficient of variation ( = mean of the 10 individual CVs; **Fig. S2b**); Grand mean: mean of the 10 individual means, each obtained as the mean of the bouts' means (**Table S5**); CVb: between-individual CV; 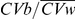: between-individual to within-individual CV ratio; ranges in parentheses.

MANOVA showed that bouts' mean acoustic properties were significantly different as a whole in different individuals (Wilks test, effect df = 135, F = 2.0, p<0.05). Nonetheless, a significant variation was found only in tonal rate, tonal fundamental frequency, and tonal fundamental frequency measured at the final (F) portion of each tonal segment (univariate one-way ANOVAs: df = 9; p<0.05 after Holm-Bonferroni correction, **Table 2**). Both within-individual and between-individual variability of all acoustic properties were relatively high (*CVw* = 1.6–300; *CVb* = 8.3–118, respectively; **Table 1**, **Fig. S2b**). The fundamental frequency of tonal segments was both the least variable acoustic property (*CVb*, 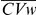 and 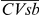; **Table 1**, **Fig. S2**), and the only one with a 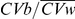 ratio >1.0 (**Table 1**).

**Table 2:** Univariate one-way ANOVAs of the mean bouts' values amongst individuals, for each acoustic properties.

Acoustic property	*n. bouts*	*N*	*F* (df 9)	*p-value*
Bout duration (s)	7.8±5.0	78	2.8	0.007
Number of pulses per bout	7.8±5.0	78	3.1	0.004
Number of tonal segments per bout	7.8±5.0	78	1.3	0.240
Pulse rate (1/s)	7.7±4.8	77	2.5	0.014
Tonal rate (1/s)	7.7±4.8	77	3.7	0.001*
Pulse duration (ms)	7.4±5.2	74	2.4	0.019
Pulse peak frequency (Hz)	7.4±5.2	74	1.3	0.246
Tonal duration (ms)	7.6±5.0	76	1.7	0.115
Tonal fundamental frequency (Hz)	7.6±5.0	76	8.8	0.000*
Tonal fundamental frequency I	7.6±5.0	76	3.1	0.003
Tonal fundamental frequency C	7.6±5.0	76	2.2	0.034
Tonal fundamental frequency F	7.5±5.0	75	4.9	0.000*
Tonal frequency modulation (C-I)	7.6±5.0	76	2.2	0.035
Tonal frequency modulation (F-C)	7.5±5.0	75	0.5	0.834
Tonal frequency modulation (F-I)	7.5±5.0	75	2.7	0.009
Pulse-pulse interval (ms)	5.7±5.0	57	1.5	0.193
Pulse-tonal interval (ms)	6.4±5.5	64	1.1	0.382
Tonal-pulse interval (ms)	5.1±3.4	51	1.7	0.124
Tonal-tonal interval (ms)	2.7±1.5	27	0.3	0.958

*statistically significant p-values (α = 0.05), after Holm-Bonferroni correction for multiple comparisons; *n. bouts*: mean and SD of the number of bouts contributed by the 10 individuals to the measured variate (all individuals differently contributed to each measured variate); *N*: number of bouts recorded for the measured variate amongst all individuals.

The correlative analysis among the bouts' acoustic properties (individual means of bouts' mean properties) revealed statistically significant constraints and trade-offs between rate or frequency and duration, between frequency and rate of different types of units, and between frequency and time intervals (alpha level 0.05, **Table S3**). The observed significant constraints between rate or frequency and duration, or between frequency and rate of different types of units, included the negative correlations between bout duration and tonal rate; between bout duration and the fundamental frequency of both the final and initial portions of tonal segments; between tonal duration and both tonal rates and the fundamental frequencies of the final portions of tonal segments; and between tonal frequency modulation (C-I and F-I) and both pulse duration and pulse rate. The observed significant constraints between frequency and intervals included the positive correlation between tonal frequency modulation (F–I) and the tonal–pulse interval. No significant correlation was found between size and any of the studied acoustic properties (**Tables S2**, **S3**).

10 behavioural acts were recognised as simultaneous or contiguous to acoustical emissions (**Table 3**; **video S1**). In particular, D1,2 and GAP were typical mudskipper aggressive visual displays. Aggressive sequences were therefore characterised by the combined use of acoustic and visual displays.

**Table 3:** Descriptions of the aggressive behaviours examined in the territorial owner during the dyadic contests, that were simultaneous or contiguous to acoustic emissions.

*D1,2*	Aggressive display of the first and second dorsal fins [25]
*APP*	Directed and rapid movement (“tripod” locomotion; [78]) towards the cage containing the intruder
*ENT*	The fish presses the snout against the cage, in an apparent attempt to reach the intruder
*GAP*	Head slightly lifted, mouth wide open, hyoid depressed and extension of branchiostegal membranes (gaping; [25]), darkening of body colouration
*SLT*	The fish draws away from the cage, heading towards the defended shelter
*TUR*	The fish turns around the cage where the intruder is confined, apparently trying to reach the intruder

Video-acoustical recordings were also examined in slow-motion. Immediately before the emission of each pulse, the head was slightly lifted, and during pulse emission the fish made a short, rapid and downwardly directed vertical movement of the mandible (during gaping), or of the whole head (closed mouth). No movements were ever observed during tonal sounds. The head was never in contact with the substrate during vocalizations. Two specimens (one male and one female), which were euthanised and dissected, lacked a gas bladder.

## Discussion

A comparative analysis between the main call properties of mudskippers and 19 other soniferous gobioid species (**Table 4**) immediately recovers clear affinities in terms of acoustic patterns. The general acoustic structure of the mudskippers' calls, that is a combination of pulsatile and tonal elements characterised by low dominant frequencies (approximately 100 Hz), closely corresponds to the typical pattern found in known soniferous gobioids, that is either a pulsatile or a mixed (tonal plus pulsatile elements) pattern, and peak/dominant frequencies comprised between 80 and 200 Hz (e.g. *Padogobius martensii*, junior syn. of *P. bonelli* (Bonaparte, 1846), [43]; **Table 4**). In a parsimonious scenario, the occurrence of similar patterns of acoustic emissions in several gobioid genera, including a member of the basal family Odontobutidae ([38], [44]; **Table 4**), and the wide geographic distribution of these species in different aquatic habitats (**Table 4**) both suggest (1) a fundamental similarity of the unknown sound production mechanism and (2) that aquatic acoustic communication is a plesiomorphic trait in gobioids.

**Table 4:** Comparative overview of sound production in gobioid fishes.

Species	Call structure (mean peak or dominant frequency)	Context	Geographic distribution	Habitat type	References
*Pomatoschistus canestrinii* (Ninni, 1883)	Pulsatile (130 Hz)	A, P	Med	B	[57]
*P. minutus* (Pallas, 1770)	Pulsatile (100 Hz)	P	EA, Med, BS	B, M	[55]
*P. marmoratus* (Risso, 1810)	Pulsatile (120 Hz)	P	Med, BS	B	[79]
*P. pictus* (Malm, 1865)	Pulsatile (80–200 Hz)	A, C, P	Med	M	[39], [54]
*Knipowitschia panizzae* (Verga, 1841)	Pulsatile (190 Hz)	P	Med	B	[79]
*K. punctatissima* (Canestrini, 1864)	Pulsatile (130 Hz)	P	Med	F	[80]
*Padogobius bonelli* (Bonaparte, 1846)	Mixed^a^ (180 Hz)	A, P, C	Med	F	[80]
*P. nigricans* (Canestrini, 1867)	Tonal (110 Hz)	C	Med	F	[81]
*Gobius paganellus* Linnaeus, 1758	Tonal (100 Hz)	A, C	EA, Med, BS	B, M	[57]
*G. cobitis* Pallas, 1814	Pulsatile (90 Hz)	A, C	EA, Med, BS	B, M	[57]
*G. niger* Linnaeus, 1758	Pulsatile (100 Hz)	A, C	EA, Med, BS	B, M	[57]
*Zosterisessor ophiocephalus* (Pallas, 1814)	Pulsatile (220 Hz)	A, C	Med, BS	B, M	[57]
*Proterorhinus marmoratus* (Pallas, 1814)	Tonal (70–130 Hz)	C	Med, BS, CS	F	[82]
*Bathygobius soporator* (Valenciénnes, 1837)	Pulsatile (145 Hz)	C	Med, EA, WA	B, M	[83]
*B. fuscus* (Rüppell, 1830)	Pulsatile (120 Hz)	C	IWP	B, M	[84]
*B. curacao* (Metzelaar, 1919)	Pulsatile (100–200 Hz)	C	WA	B	[85]
*Gobiosoma bosc* (Lacepéde, 1800)	Clicks (1–5 kHz)	C	WA	B, M	[86]
*Odontobutis obscura* (Temminck & Schlegel, 1845)	Pulsatile (300 Hz)	C	China, Japan, Korea	F	[38]
*Neogobius melanostomus* (Pallas, 1814)	Pulsatile (180 Hz)	C	BS, CS	F	[87]

a the call is composed by tonal and pulsatile elements; A = aggressive; B = brackish; BS = Black Sea; C = courtship; CS = Caspian Sea; EA = Eastern Atlantic; F = freshwater; IWP = Indo-West Pacific region; M = marine; Med = Mediterranean; P = prespawning; WA = Western Atlantic.

These observations support the guiding “exaptation hypothesis” in mudskippers. The gobioid structure of *P. septemradiatus* vocalizations both suggests that during their eco-evolutionary transition to an amphibious lifestyle mudskippers retained ancestral acoustic traits, and that other oxudercine gobies are soniferous. In an adaptive perspective, our results also suggest that in amphibious gobies eco-ethological adaptations likely preceded rather than followed new adaptations to terrestrial conditions.

In fact, mudskippers may communicate acoustically inside their water-filled burrows, involving both aggressive and reproductive behaviours; nonetheless, no underwater interactions were obtained in the laboratory during this study. In this respect, the acoustic sensitivity of the closely related species *Periophthalmus barbarus* (Linnaeus, 1766), measured under water (100–900 Hz [42]) and inferred in behavioural studies made out of water in *Periophthalmus koelreuteri* (Pallas, 1770) = jun syn. of *P. barbarus* (258–651 Hz [41]) reveals a good overall correspondence with the observed peak frequencies of sound emissions.

In a wider perspective, gobioids are typically benthonic fishes, and the structure of mudskippers' sounds is also similar to other non-gobioid benthonic teleosts, such as toadfishes (Batrachoididae; [45]–[47]), blennies (Blennidae; [48]) and sculpins (Cottidae; [49], [50]), some of which have well-known sonic mechanisms associated with extremely specialised anatomical structures [47]. This suggests (1) that this peculiar sound structure might have evolved multiple times in aquatic benthonic habitats, and (2) that a benthonic lifestyle might have facilitated water-to-land transitions in mudskippers.

The mean fundamental frequency of tonal segments (1) was significantly different among individuals; (2) exhibited a relatively stereotyped nature; and (3) had a 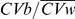 ratio >1.0, therefore showing a potential for acoustic communication, and suggesting that tonal segments act as acoustical taggers [51] and neighbour-stranger discriminants (*dear enemy effect*; [45]). In the aquatic goby *P. bonelli*, the wider scope for frequency and amplitude modulation of tonal sounds apparently improved both propagation and signal recognition [46], [52]. Acoustical tagging and dear enemy effects would be advantageous for territorial mudskipper species. Future playback and discrimination experiments could verify these hypotheses.

In general, all the examined acoustic properties of *P. septemradiatus* would be classified as *dynamic* [53], due to their high level of within-individual variation, a feature observed also in other gobies [54], [55], which nonetheless does not imply low repeatability [53]. The contiguous or simultaneous emission of sounds with visual aggressive displays that were previously described in oxudercine species (*Periophthalmus* sp.: [25]), such as gaping and dorsal fin erection, suggests the use of multimodal visual-acoustic communication, as hypothesised in other gobies [56].

Sonic organs are unknown in gobioids. In *P. septemradiatus*, the temporal association of pulsatile and tonal units, which never overlapped, suggests either a unique or two synchronised sonic mechanisms. In the first, most parsimonious hypothesis, the adjustable frequency of a sonic muscle would produce both pulses and tonal segments [57]. Significant correlations between size and acoustic properties were observed in many soniferous fishes (e.g. Triglidae [58]; Mormyridae [59]; Osphronemidae [60]; Mochokidae [61]; Pomacentridae [62]; Batrachoididae [63]); and Gobiidae [64]). Nonetheless, *P. septemradiatus* is not an isolated exception: no correlation was found between body size and acoustic properties in either aggressive and courtship sounds also in the freshwater goby *Padogobius bonelli* [65], whose structural acoustic properties closely resemble those of *P. septemradiatus*. Therefore, unless the mechanisms of sound emission are clarified in gobioids, no general conclusion on the relationship between body size and acoustic properties can be drawn.

Several teleosts apparently use a gas bladder to amplify the vibrations produced by sonic muscles [46]; nonetheless, not unlike other mudskipper species [66], *P. septemradiatus* lacks a gas bladder. Mudskippers may also be able to use the gas bubble retained during air-gulping [67] as a resonant structure; nonetheless, during our observations sounds were also produced with apparently deflated opercular chambers. Stridulatory mechanisms are also improbable, since these sounds generally have much higher fundamental frequencies [46]. The observed condition is compatible with a sonic muscle utilising a part of the body as a sound transducer (e.g. the pectoral girdle; [46], [49]). Our correlation analysis of the acoustic properties supports this hypothesis: the observed constraints and trade-offs are consistent with a more rapid onset of muscular fatigue induced by higher rates, higher frequencies, and shorter intervals between units. Other correlations can be related to individual motivation [46], such as the negative correlation between pulse rate and the pulse–tonal interval; and the positive correlations between tonal rate and the fundamental frequency of the final portion of tonal segments.

Substrate-borne vibrations (e.g. S waves) could reach the otic capsule through the pectoral fins and girdle (e.g. through posttemporal bones; [8]), or even be perceived through the neuromasts of the head [68] and of the caudal fin (the lateral line system is greatly reduced in mudskippers [8], [69]).Sound production and reception while out of water would depend on the transmitting media. The hydrophone inserted into the exposed wet mud measured compression waves transmitted in the near field through the superficial layers of water-saturated mud [49], [70], [71]. Whatever the sonic mechanism, during sound emissions the particle displacements of the sediment surface or superficial layers act as sources of strictly coupled [72] compression and seismic waves, which propagate both inside the substrate, air, and capillary water; and at different types of physical interfaces [49], [73]. These waves could be perceived either at surfaces, or within one of the component media [73], [74], or both. In fact, acoustic communication at the substrate's surface was suggested in *C. bairdi* [49], since Rayleigh waves produced by sonic behaviours were less attenuated in the near field than compression waves. Acoustic communication at the substrate's surface was also suggested in aquatic gobies [56]. In fact, the acoustic “thumps” emitted by sand gobies and their associated head movements [54], [56] resemble the mudskippers' pulsatile components and associated movements.

Rather than pressure waves, sounds are possibly perceived as particle displacements at the substrates' surface also by mudskippers. Therefore, we corroborated our results obtained with the buried hydrophone with supplementary recordings of artificially generated tonal and pulsed sounds, measured in terms of both pressure and particle velocity (**Supplementary text S1**; **Figs. S4**, **S5**). Similar to the results of Lugli & Fine [75] on stream ambient noise and sounds produced by *P. bonelli* (transmitted through water), in our trials the properties of substrate-transmitted sounds were similar both in terms of pressure and particle velocity perturbations (**Figs. S4**, **S5**), suggesting a strict relationship between compression waves and particle displacements. Energy spectra showed that both compression waves and particle displacements were efficiently propagated at distances of few cm.

The problem of the discrimination amongst signal components transmitted and received through different physical media and their interfaces in amphibious fishes opens future research perspectives.

## Materials and Methods

### Ethics statement

All laboratory protocols and ethological observations followed the guidelines provided by the Italian laws on the use of animals for experiments (Decreto Legislativo n. 116, 1992). According to this definition, approval from any institution was not necessary. In fact, the present study is an observational work, with a minimum degree of manipulation of the experimental animals. All the specimens were reared in the laboratory using all the methods and equipments to recreate the best biotic and abiotic environmental conditions. One male and one female *P. septemradiatus* were anesthetised in MS222 and euthanised with cold at −20°C to conduct anatomical observations.

### Experimental apparatus and design

Preliminary recordings in 5 communal tanks (**Fig. S3**) demonstrated the presence of acoustic communication during males' competitive feeding. Sounds could be recorded with a hydrophone (B&K 8103 Naerum, Denmark; sensitivity −210 dB re 1 µPa) inserted into the wet mud and connected to a conditioning amplifier (B&K 2626 Naerum, Denmark) and to a portable digital audio tape recorder (DAT: Sony D7 Park Ridge, NJ, USA).

Therefore, standardised protocols were designed to observe dyadic male-male encounters and record the associated sound production. Single males were isolated in experimental tanks (same size and equipment of the communal tanks), but only a single shelter made of slate pieces or terracotta was provided, which was rapidly occupied. A single hydrophone was inserted into the mud at a depth of 2–5 cm, in front of the opening of the resident's shelter and within an acceptable range of the attenuation distance from the source of possible vocalizations [76], being connected both to the DAT and a digital video-camera (25 fps; Canon MV400 New York, NY, USA), coupling video-acoustical recordings. The recording volume was manually set between levels 4 and 6. Each device was electrically insulated. Experiments started after at least 48 hrs, allowing acclimatisation, territorial establishment and residency. Each resident was then exposed to a male intruder of comparable size, caged in a cylindrical metallic net (diameter: 12 cm; height: 20 cm; mesh size: 1 cm), closed on top and fixed into the substrate at ∼15 cm from the shelter's opening. Video recordings allowed to select calls visibly produced by residents only. During recordings of dyadic contests no food was provided, to prevent competitive feeding behaviours and avoid possible masking effects of feeding on agonistic vocalisations. Temperature was maintained constant within and across the experimental tanks (**Supplementary text S1**).

Behavioural observations were conducted with the aid of a small window created within an opaque divisor placed between the tank and the observer, in order to reduce visual interference.

### Sound and data analysis

The aggressive responses of the focal animal (resident) prevalently took place within a restricted area comprised between the cage and the shelter, at a few cm from the hydrophone.

Sounds were analysed in real time (SASLab Pro© Avisoft Bioacoustics Berlin, Germany; window type: hamming, FFT: 256, frame: 100, bandwidth: 20 Hz, resolution: 16 Hz, overlap: 87.5%; **Fig. S1**). Analogical signals were digitalised (1,500 Hz sampling) and acoustic components which were not present in the recorded fish sounds (band: 30–500 Hz) were band-pass filtered, in order to eliminate sources of disturbance and distortion for the mudskippers' waveforms. Only signals with higher signal to noise ratios were analysed.

The recorded calls of 10 individuals were resolved into 78 “complex bouts” by defining a minimum time gap between two subsequent sound units (5 s). Bouts were then broken down into pulsatile and tonal units [77], and several acoustic properties were defined, measured and analysed (STATISTICA v 7.0© StatSoft Tulsa, OK, USA; **Table S1**, **S2**). In particular, tonal frequency modulation was quantified as the differences between the frequency of initial (I) and central (C); initial and final (F); and central and final portions of tonal segments, each portion corresponding to 4 cycles, randomly taken from each portion of sound [57].

To estimate whether the observed acoustic properties varied significantly amongst individuals, the means of the bouts' values of each individual were logarithmically transformed to conduct a one-way MANOVA to test for multivariate difference amongst individuals; and 19 one-way ANOVAs with Holm-Bonferroni corrections, to specifically test for each acoustic property (**Table S4**). Mean within-bout (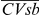), mean within-individual (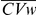), and between-individual (*CVb*) coefficients of variation (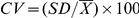; untransformed data [54]) were respectively calculated: 1) as the mean of the bouts' CVs (*CVsb*; **Fig. S2a**); 2) as the mean of the individual CVs, obtained from the bouts' means per individual (*CVw*; **Fig. S2b**); and 3) from the 10 individual means (**Table S5**), each obtained as the mean of the bouts' means; i.e. *CVb* = (*SD/Grand mean*)·100; **Table 1**. 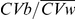 ratios were utilized as a measure of relative variability among individuals ( = *CVb/CVw* in [54]).

Video-acoustical recordings of mudskipper behavioural interactions allowed the identification and description of behavioural acts that were contiguous or simultaneous to acoustical emissions.

To formulate first hypotheses on the emission mechanism, video-acoustical recordings were examined in slow-motion, and a correlative analysis was conducted amongst individual mean acoustic properties and individual body size, after logarithmic transformation.

## Supporting Information

Figure S1 Temporal organisation of a mudskipper call and some acoustic properties. *p* pulse; *p+t* fused pulse and tonal segment; *t* tonal segment; *PD* pulse duration; *PPI* pulse-pulse interval; *TD* tonal duration; *TPI* tonal-pulse interval; *TTI* tonal-tonal interval.
(TIF)

Figure S2Boxplots of the within-bout (a: *CVsb*) and within-individual (b: *CVw*) coefficients of variation. *CVsb* are the coefficients of variation of acoustic properties of the sound elements measured in each of the 78 bouts (mean values = 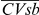). *CVw* are the coefficients of variation of the mean acoustic properties of the bouts of each of the 10 individuals (mean values = 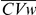). Boxes indicate the middle 50% of the distribution (interquartile range); whiskers indicate minimum and maximum values; horizontal lines are median values. *BD* bout duration; *NP* number of pulses; *NTS* number of tonal segments; *PD* pulse duration; *PPF* pulse peak frequency; *PPI* pulse-pulse interval; *PR* pulse rate; *PTI* pulse-tonal interval; *TD* tonal duration; *TFM* tonal frequency modulation (*I* initial portion of the tonal segment; *C* central portion of the tonal segment; *F* final portion of the tonal segment); *TFF* tonal fundamental frequency; *TPI* tonal-pulse interval; *TR* tonal rate; *TTI* tonal-tonal interval (see also **Table 1**).(TIF)

Figure S3 Layout of the housing terraria (community tank). *FP*: polyurethane foam panel; *h/T*: hygrometer's and thermostat's probes; *IR*: thermostated heating lamps; *Md*: mud; *P*: pool (non toxic plastic bowl); *T*: thermometer; *z*: three parallel zones separated by wooden logs and flat slate pieces to reduce aggressive interactions.
(TIF)

Figure S4 Spectrograms, power spectrum and waveforms of tonal artificial sounds acoustically similar to the tonal segments of the calls of *P. septemradiatus*, synchronously recorded in terms of pressure (a, b and c) and particle velocity (d, e and f); sounds were produced as a descending scale from 500 Hz at third octave steps (hamming FFT: 512, frame: 100, bandwith: 10 Hz, resolution: 8 Hz, overlap: 93.75%); for the power spectra, amplitude on a linear scale of 100 mV per division (arbitrary units).
(TIF)

Figure S5 Spectrograms, power spectrum and waveforms of pulsed artificial sounds acoustically similar to the pulsatile elements of the calls of *P. septemradiatus*, synchronously recorded in terms of pressure (a) and particle velocity (b); see **Figs. S4** for more details.
(TIF)

Text S1 The species studied and housing conditions. Tonal and pulsed artificial sounds through the substrate, recorded as particle displacements and pressure waves. Supplementary references.
(DOCX)

Video S1 A complex bout emitted by a male of *P. septemradiatus*. Left panel: video recording; the fish, which is a resident territorial owner, is oriented towards the cage containing the intruder (on the right, not visible). The oscillogram (top right panel) and spectrogram (bottom right panel) of the emitted bout show a train of pulses followed by a tonal segment. Acoustical and video recordings are synchronised. Note the rapid downward movements of the head made during the pulse emissions, and the behaviours preceding and following the vocalisation (dorsal fins' display and jump, respectively). During the sequence, the mudskipper is also retracting its eyes into the dermal cups positioned below the orbits (“blinking”), to clean and moisten the eye surface while out of water.
(MPG)

Table S1 Descriptions of the acoustical properties of bouts and sound units.
(DOCX)

Table S2 Size, number of acoustic bouts and sound units of the recorded resident individuals.
(DOCX)

Table S3 Pearson correlation coefficients (* = *p*<0.05) of the relationships amongst the individual means of each acoustic property (n = 10 specimens).
(DOCX)

Table S4 Mean acoustic properties of bouts per individual.
(DOCX)

Table S5 Individual means.
(DOCX)
