# Supplementary figures and images for: Acoustic Communication at the Water's Edge: Evolutionary Insights from a Mudskipper
Source: PLoS One. 2011 Jun 28;6(6):e21434. doi: 10.1371/journal.pone.0021434 (PMC3125184; doi:10.1371/journal.pone.0021434)

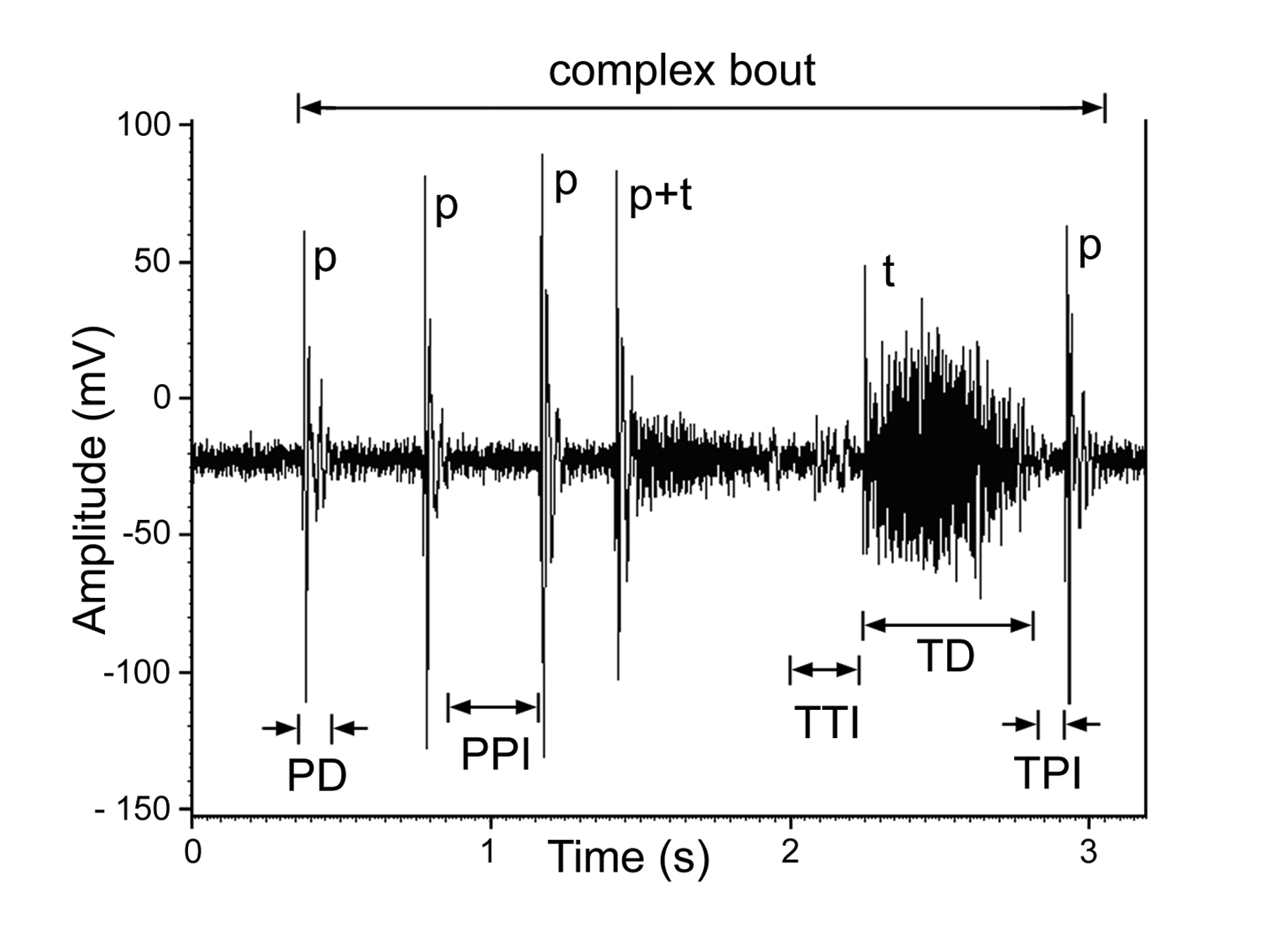

Supplement: Figure S1 — Temporal organisation of a mudskipper call and some acoustic properties. p pulse; p+t fused pulse and tonal segment; t tonal segment; PD pulse duration; PPI pulse-pulse interval; TD tonal duration; TPI tonal-pulse interval; TTI tonal-tonal interval. (TIF) [file pone.0021434.s001.tif]

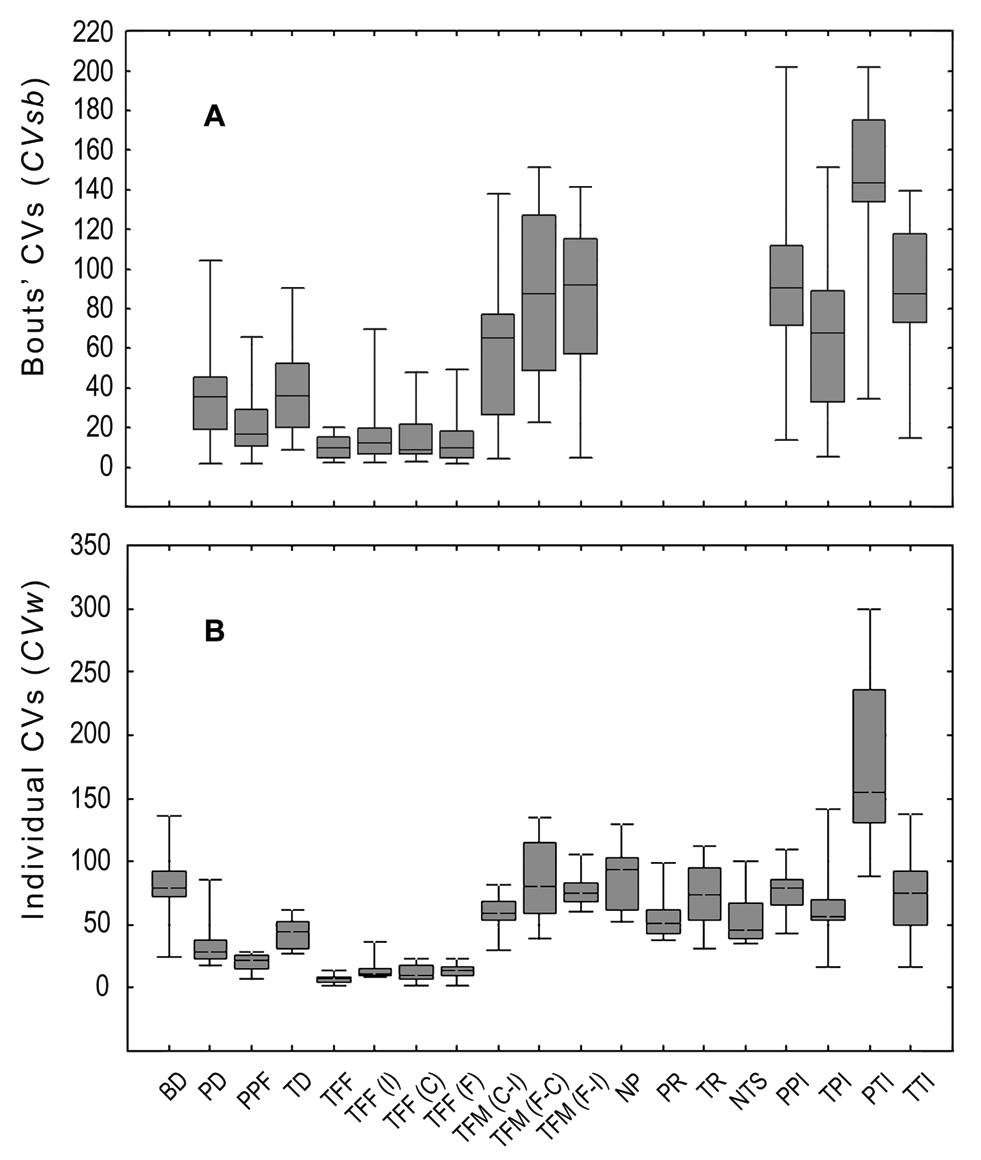

Supplement: Figure S2 — Boxplots of the within-bout (a: CVsb) and within-individual (b: CVw) coefficients of variation. CVsb are the coefficients of variation of acoustic properties of the sound elements measured in each of the 78 bouts (mean values = ). CVw are the coefficients of variation of the mean acoustic properties of the bouts of each of the 10 individuals (mean values = ). Boxes indicate the middle 50% of the distribution (interquartile range); whiskers indicate minimum and maximum values; horizontal lines are median values. BD bout duration; NP number of pulses; NTS number of tonal segments; PD pulse duration; PPF pulse peak frequency; PPI pulse-pulse interval; PR pulse rate; PTI pulse-tonal interval; TD tonal duration; TFM tonal frequency modulation (I initial portion of the tonal segment; C central portion of the tonal segment; F final portion of the tonal segment); TFF tonal fundamental frequency; TPI tonal-pulse interval; TR tonal rate; TTI tonal-tonal interval (see also Table 1 ). (TIF) [file pone.0021434.s002.tif]

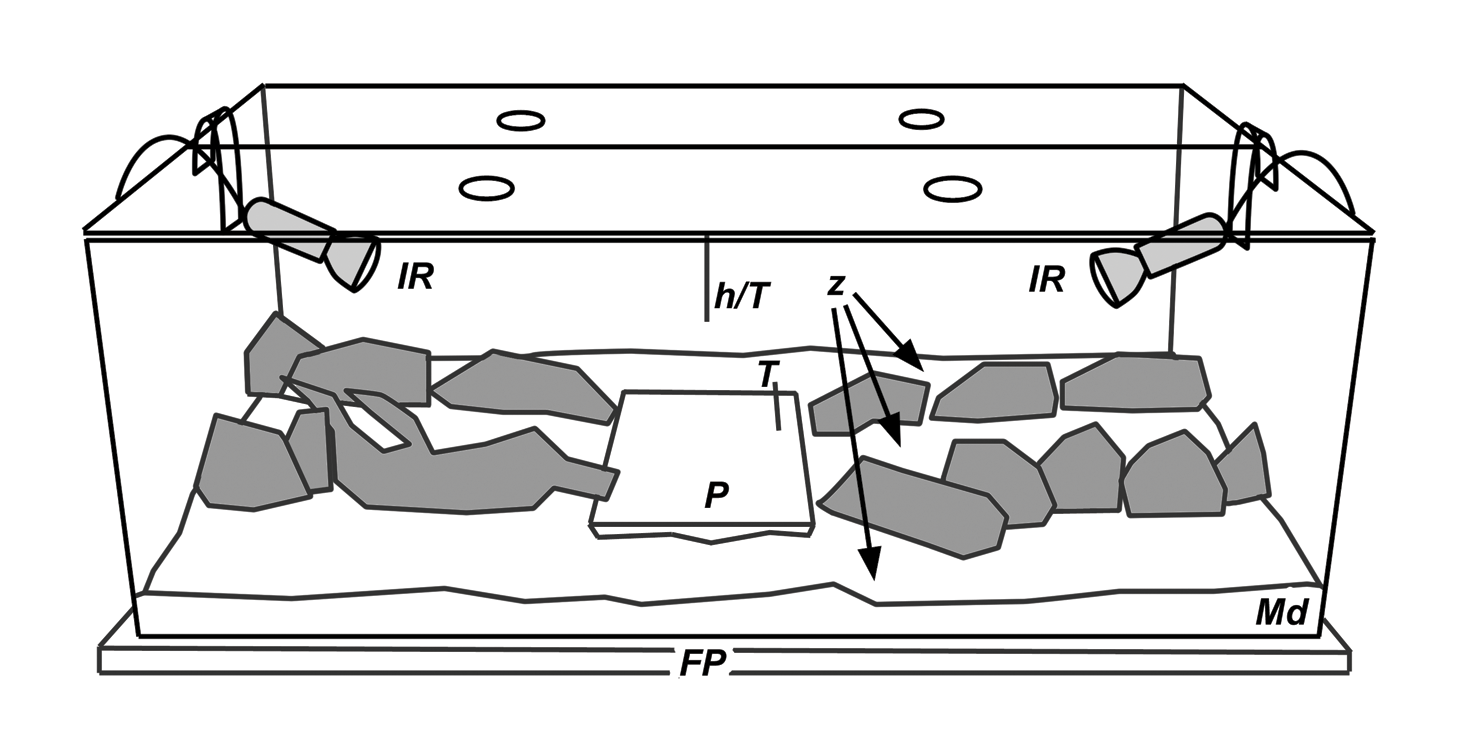

Supplement: Figure S3 — Layout of the housing terraria (community tank). FP: polyurethane foam panel; h/T: hygrometer's and thermostat's probes; IR: thermostated heating lamps; Md: mud; P: pool (non toxic plastic bowl); T: thermometer; z: three parallel zones separated by wooden logs and flat slate pieces to reduce aggressive interactions. (TIF) [file pone.0021434.s003.tif]

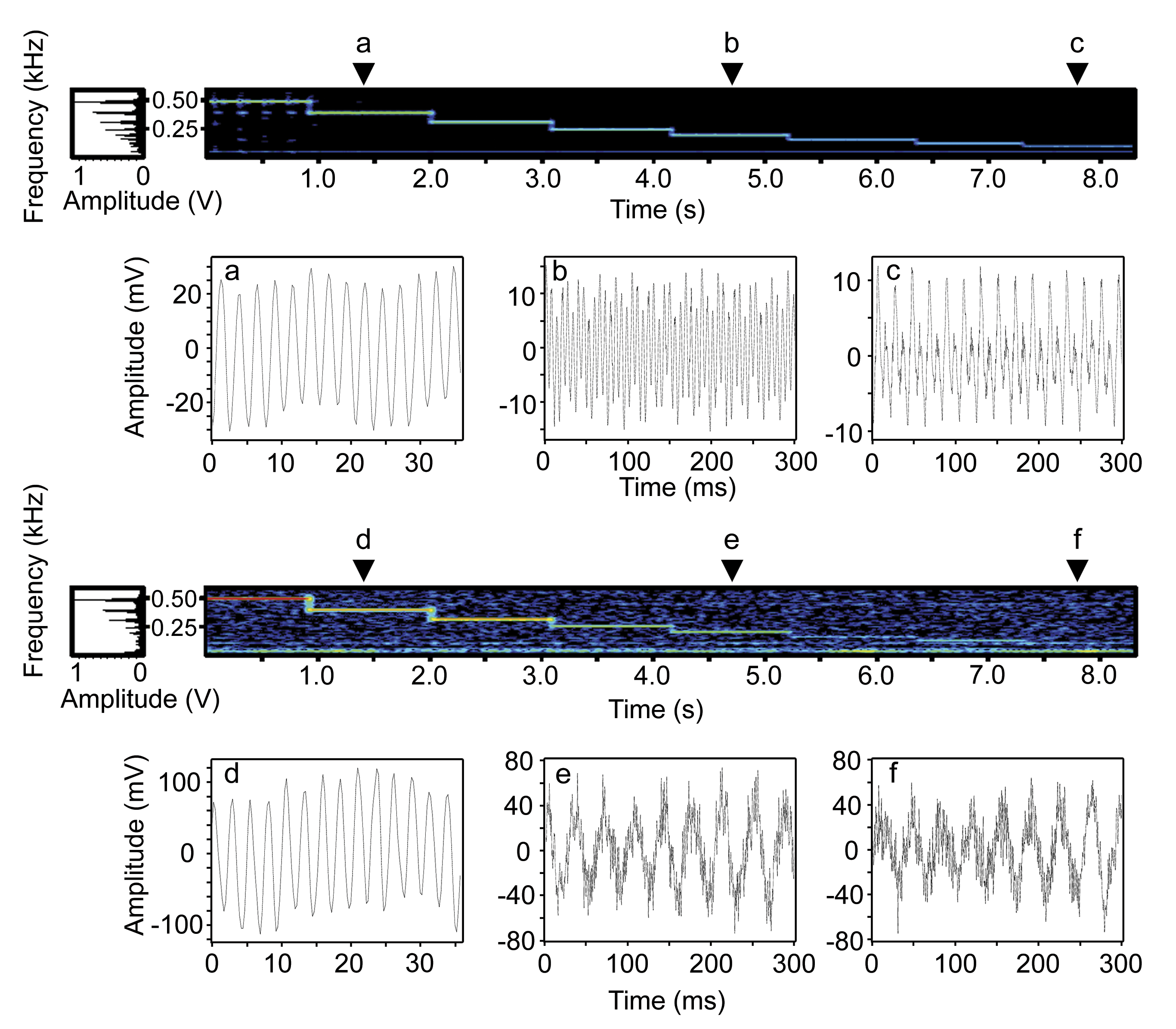

Supplement: Figure S4 — Spectrograms, power spectrum and waveforms of tonal artificial sounds acoustically similar to the tonal segments of the calls of P. septemradiatus, synchronously recorded in terms of pressure (a, b and c) and particle velocity (d, e and f); sounds were produced as a descending scale from 500 Hz at third octave steps (hamming FFT: 512, frame: 100, bandwith: 10 Hz, resolution: 8 Hz, overlap: 93.75%); for the power spectra, amplitude on a linear scale of 100 mV per division (arbitrary units). (TIF) [file pone.0021434.s004.tif]

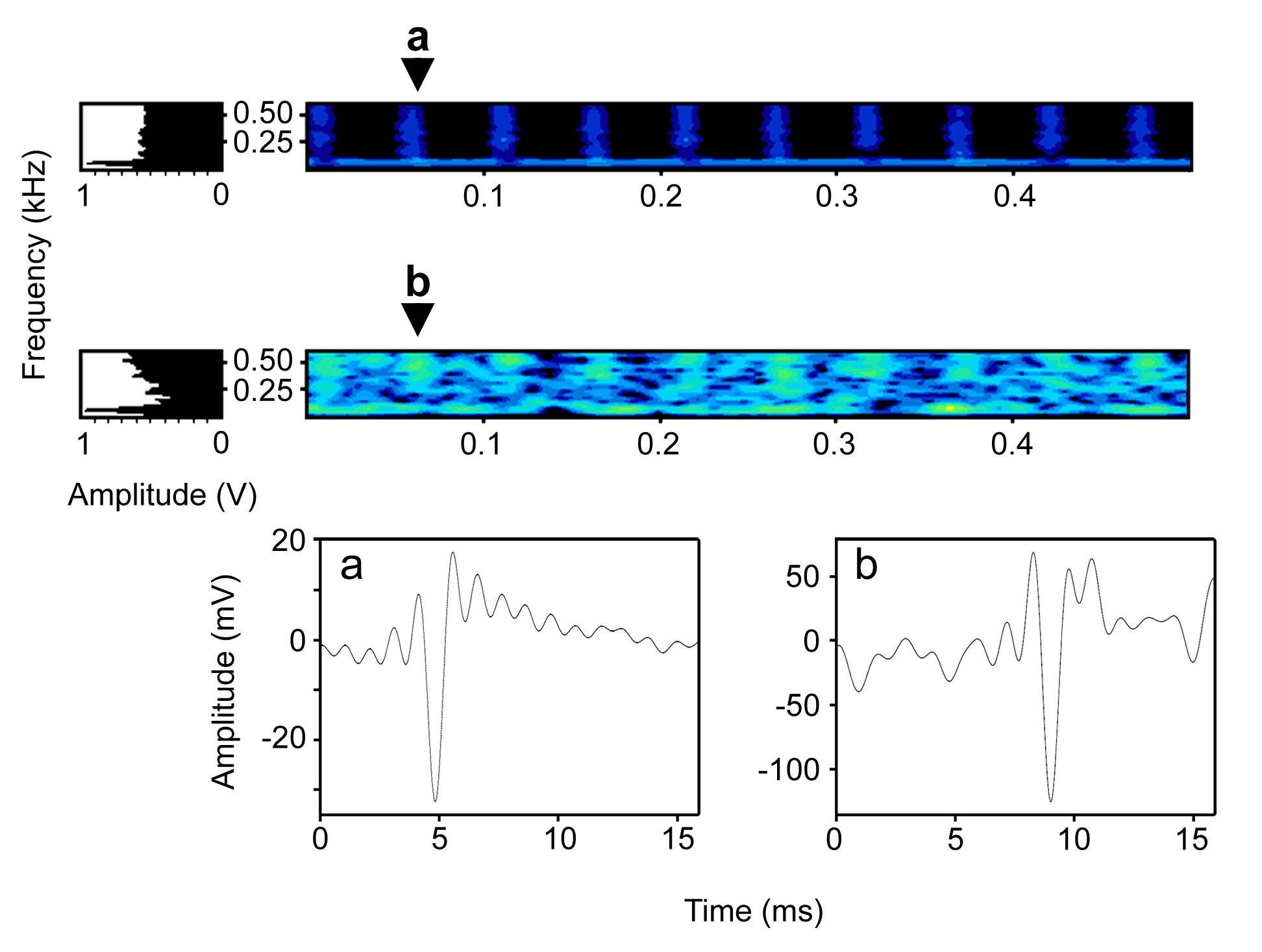

Supplement: Figure S5 — Spectrograms, power spectrum and waveforms of pulsed artificial sounds acoustically similar to the pulsatile elements of the calls of P. septemradiatus, synchronously recorded in terms of pressure (a) and particle velocity (b); see Figs. S4 for more details. (TIF) [file pone.0021434.s005.tif]
