# Supplementary material for: Acoustic Communication at the Water's Edge: Evolutionary Insights from a Mudskipper
Source: PLoS One. 2011 Jun 28;6(6):e21434. doi: 10.1371/journal.pone.0021434 (PMC3125184; doi:10.1371/journal.pone.0021434)
Supplement: Text S1 — The species studied and housing conditions. Tonal and pulsed artificial sounds through the substrate, recorded as particle displacements and pressure waves. Supplementary references. (DOCX) [file pone.0021434.s006.docx]

Supplementary Text S1

**The species studied and housing conditions**

*Periophthalmodon septemradiatus* (**Fig. 1**) is distributed throughout the Indo-Malayan region, from the Ganges delta to insular Malaysia (Murdy 1989) and Viet Nam. It lives in forested supratidal habitats, in proximity of tidally influenced small streams and pools with very low salinities ([88,89]; G. Polgar *pers. obs.*).

30 specimens of *P. septemradiatus* were imported from Viet Nam in December 2008 through a local dealer and are presently still reared in our laboratories in Venice to conduct further experiments.

Fishes were measured to the nearest tenth of mm (standard length: SL), sexed and separately placed in 8 terraria (height: 35 cm, length: 100 cm; depth: 50 cm; **Fig. S3**). Sexes in *P. septemradiatus* are strikingly dimorphic, with mature males’ first dorsal fins reaching the point of insertion of second dorsal fins, while females’ ones are atrophic (Murdy 1989).

A layer of 5-10 cm of highly organic intertidal mud was used as substrate. The mud was collected in an only slightly polluted area of the Venice lagoon (Italy), stirred and washed several times, and then treated for three days with a 6.5%_wt_ solution of oxygen peroxide (Carlo Erba Reagents Milano, Italy) to reduce the risk of possible contaminants and pathogens. The mud was then saturated with artificial (Ocean fish, Prodac® Padova, Italy) brackish water and contaminated with sediments collected in a Malayan mangrove forest, to facilitate colonisation by decomposing bacteria, fungi, algae and other benthic microbes. Mud mean interstitial salinity was adjusted at 10–15 (hand-held refractometer ©ATAGO Tokyo, Japan; [90]), and its mean water content at ~30–60% wt H_2_O; 3 mud samples in 4 tanks were weighed before and after dehydration at 65°C to constant weight, and mud water content was calculated as: ((wet weight - dry weight)/wet weight)· 100. Mud physical conditions were maintained by periodically replacing the evaporated water.

Mudskippers are highly territorial species: only 3-4 specimens were put in each tank, and barriers (wooden logs, flat slate pieces) were disposed to divide each tank into three parallel zones and reduce aggressive interactions (**Fig. S3**). A shallow pool (~32°C; salinity 5–10) was positioned in the middle of the tank (nontoxic material for human huse), to allow symmetrical access to all individuals. Each paludarium was closed on top to prevent escapes and maintain both humid and hot air and substrate conditions.

Air temperature and humidity were kept constant (33±1.8°C; ~90% RH) by two thermostated heating lamps per tank (ZOO MED, 100 W San Luise Obispo, CA, USA) connected to an external thermostat (Resun TS-600 Guangdong Province, China, sensitivity: ±1°C). The lamps’ position (**Fig. S3**) allowed for different air temperatures inside the tank during winter (total range ~ 4°C), while during spring and summer (when video-acoustical recordings were made) differences inside terraria were barely measurable.

Illumination was provided by neon tubes (one tube per tank: SYLVANIA F18 W/154 T8 Danvers, MA, USA), and attenuated to recreate natural conditions. Fish were fed on fish pellets and chironomid larvae. Polyurethane foam panels were used to soundproof each tank from ground.

**Tonal and pulsed artificial sounds through the substrate, recorded as particle displacements and pressure waves.**

We corroborated our results by recording artificial tonal and pulsatile sounds transmitted through the substrate with a buried underwater pressure-velocity probe. The utilised underwater probe did not allow to make measurements along vertical directions. Nonetheless, we may preliminarily assume that the particle displacements generated by the unknown mudskipper emission mechanism and propagating from the surface to the buried hydrophone (that we recorded as compressive waves) along a diagonal or vertical direction are behaving in the same general way. The synchronous recording of the temporal changes of pressure (*p*) and particle velocity (*u*) into the mud and along a horizontal direction between the probe and the sound source allowed to compare the spectral and temporal characteristics (energy distribution, wave forms) of both compressive waves and particle displacements in the same experimental conditions (see material and methods, main text).

The underwater acoustic pressure-velocity probe (Mk.2 Acoustech © outer Ø : 4.3 cm, Philadelphia, PA, USA), contained two built-in units: a piezoelectric omni-directional hydrophone (sensitivity: -203.1 dB re: 1 V/μPa) to measure acoustic pressure (*p*); and a bidirectional geophone (sensitivity: 10.5) to measure particle velocity (*u)* along one axis. The single probe (recording *p* and *u* at the same time) was inserted into the mud at a depth of 2‒5 cm, as in the protocol adopted to record the mudskippers’ calls. A tripod and a connected iron support were used to anchor the probe into the mud, and orient the piezoelectric disk towards the sound source. The sound source was provided by another piezoelectric disk connected to an audio generator (MINIRATOR MR1, analog audio generator, NTI Neutrik Test Instruments, Schaan Liechtestein) and inserted under the mud surface at few cm from the probe, thus simulating the experimental conditions (see materials and methods, main text). The probe was connected to an external, battery-operated two-channel preamplifier (Acoustech© Nebury Park, CA, USA). Separate output jacks of the preamplifier for pressure and velocity were connected to a portable Digital Audio Tape (DAT, Sony D7, NJ USA).

A series of pulsatile sounds of about 400 Hz and frequency modulated tonal sounds (a descending scale of tonal sounds from 500 Hz, generated at third octave steps) was produced. Sounds were exported to a pc (sampling rate 4000 Hz) and analysed with AVISOFT© (Berlin, Germany). Recordings were band-pass filtered between 90 Hz and 1 kHz, to avoid the geophone’s two resonance peaks [75].

A comparison between the energy and temporal properties measured as *p* and *u* (**Figs. S4**, **S5**), shows that both energy distributions and wave forms are similar (i.e. **Figs. S4a**,**b**,**c**, vs. **Fig.S4d**,**e**,**f**, respectively; and **Fig. S5a** vs. **Fig. S5b**), although the wave form of tonal sounds recorded as particle velocity is less clear below 200 Hz (**Fig. S4f**); and the energy distribution of pulsatile sounds recorded as particle velocity is shifted to higher energies (**Fig. S5b**). However, tracks from particle velocity measures are more disturbed by the background noise, as shown by the spectrograms, making sonograms and wave forms less clear.

These results are largely consistent with those of Lugli & Fine [75], who compared the energy distribution and wave form of the sounds emitted by a freshwater goby and transmitted through the water. The generality of such results strongly supports that both particle displacements and compressive waves travelling horizontally and vertically through the surface of the wet mud and from the surface through the superficial layer (2‒5 cm) have similar acoustic properties. Although the mudskipper receptor is not known, in the reasonable hypothesis that reception occurs as particle displacements at the level of the sediment surface, it is probable that the acoustic properties of such physical perturbations are extremely similar to those ones of the recorded compressive waves.

**References**

1. Khaironizam MZ, Norma-Rashid Y (2003) First record of the mudskipper, Periophthalmodon septemradiatus (Hamilton) (Teleostei: Gobiidae) from Peninsular Malaysia. Raffles Bull Zool 51: 97–100.
2. Takita T, Agusnimar, Ali AB (1999). Distribution and habitat requirements of oxudercine gobies (Gobiidae: Oxudercinae) along the Straits of Malacca. Ichthyol Res 46: 131–138.
3. English S, Wilkinson C, Baker V (1997) Survey manual for tropical marine resources, 2^nd^ edition. Townsville: Australian Institute of Marine Science. 390 p.
