# Supplementary material for: Acoustic Communication at the Water's Edge: Evolutionary Insights from a Mudskipper
Source: PLoS One. 2011 Jun 28;6(6):e21434. doi: 10.1371/journal.pone.0021434 (PMC3125184; doi:10.1371/journal.pone.0021434)
Supplement: Table S1 — Descriptions of the acoustical properties of bouts and sound units. (DOCX) [file pone.0021434.s008.docx]

**Table S1.** Descriptions of the acoustical properties of bouts and sound units.

| Pulse rate (1/s) | ratio between the number of pulses and the bout duration |
| --- | --- |
| Tonal rate (1/s) | ratio between the number of tonal segments and the bout duration |
| Pulse duration (ms) | time from the beginning to the end of a pulse |
| Pulse peak frequency (Hz) | peak frequency of single pulses, measured from its power spectrum function |
| Tonal duration (ms) | time from the beginning to the end of a tonal segment |
| Tonal fundamental frequency (Hz) | frequency of the whole tonal segment, from the power spectrum function (first harmonic band) |
| Tonal fundamental frequency I | frequency measured at the initial (I) portion of each tonal segment, as the reciprocal of the duration of 4 cycles of the wave form |
| Tonal fundamental frequency C | frequency measured at the central (C) portion of each tonal segment, as the reciprocal of the duration of 4 cycles of the wave form |
| Tonal fundamental frequency F | frequency measured at the final (F) portion of each tonal segment, as the reciprocal of the duration of 4 cycles of the wave form |
| Tonal frequency modulation (C-I)^a^ | difference between the peak frequencies measured at the central (C) and initial (I) portions of the tonal segments |
| Tonal frequency modulation (F-C)^a^ | difference between the peak frequencies measured at the final (F) and central (C) portions of the tonal segments |
| Tonal frequency modulation (F-I)^a^ | difference between the peak frequencies measured at the final (F) and initial (I) portions of the tonal segments |
| Pulse-pulse interval (ms) | time interval from the end of one pulse to the beginning of the next one |
| Pulse-tonal interval (ms) | time interval from the end of one pulse to the beginning of the subsequent tonal segment |
| Tonal-pulse interval (ms) | time interval from the end of a tonal segment to the beginning of the subsequent pulse |
| Tonal-tonal interval (ms) | time interval from the end of a tonal segment to the beginning of the next one |

^a^Frequency modulations were reported in absolute value.
