# Supplementary material for: Acoustic Communication at the Water's Edge: Evolutionary Insights from a Mudskipper
Source: PLoS One. 2011 Jun 28;6(6):e21434. doi: 10.1371/journal.pone.0021434 (PMC3125184; doi:10.1371/journal.pone.0021434)
Supplement: Table S2 — Size, number of acoustic bouts and sound units of the recorded resident individuals. (DOCX) [file pone.0021434.s009.docx]

**Table S2.** Size, number of acoustic bouts and sound units of the recorded resident individuals.

| **Individual** | **SL (mm)** | **bouts** | **pulses** | **Tonal segments** | |
| --- | --- | --- | --- | --- | --- |
| 1 | 6.3 | 10 | 27 | 12 |  |
| 2 | 6.5 | 6 | 38 | 14 |  |
| 3 | 7.1 | 4 | 12 | 6 |  |
| 4 | 7.1 | 19 | 122 | 29 |  |
| 5 | 7.3 | 7 | 50 | 12 |  |
| 6 | 7.5 | 4 | 27 | 10 |  |
| 7 | 7.5 | 8 | 10 | 10 |  |
| 8 | 7.6 | 13 | 56 | 17 |  |
| 9 | 7.7 | 3 | 12 | 4 |  |
| 10 | 8.3 | 4 | 26 | 5 |  |
