# Supplementary material for: Acoustic Communication at the Water's Edge: Evolutionary Insights from a Mudskipper
Source: PLoS One. 2011 Jun 28;6(6):e21434. doi: 10.1371/journal.pone.0021434 (PMC3125184; doi:10.1371/journal.pone.0021434)
Supplement: Table S3 — Pearson correlation coefficients (* = p<0.05) of the relationships amongst the individual means of each acoustic property (n = 10 specimens). (DOCX) [file pone.0021434.s010.docx]

**Table S3.** Pearson correlation coefficients (* = *p* < 0.05) of the relationships amongst the individual means of each acoustic property (n = 10 specimens).

|  | *BD* | *NP* | *NTS* | *PR* | *TR* | *PD* | *PPF* | *TD* | *TFF* | *TFF (I)* | *TFF (C)* | *TFF (F)* | *TFM (C-I)* | *TFM (F-C)* | *TFM (F-I)* | *PPI* | *PTI* | *TPI* |
| --- | --- | --- | --- | --- | --- | --- | --- | --- | --- | --- | --- | --- | --- | --- | --- | --- | --- | --- |
| *NP* | *0.90 | 1.00 |  |  |  |  |  |  |  |  |  |  |  |  |  |  |  |  |
| *NTS* | 0.53 | 0.57 | 1.00 |  |  |  |  |  |  |  |  |  |  |  |  |  |  |  |
| *PR* | 0.11 | 0.44 | 0.27 | 1.00 |  |  |  |  |  |  |  |  |  |  |  |  |  |  |
| *TR* | *-0.68 | -0.61 | 0.08 | 0.14 | 1.00 |  |  |  |  |  |  |  |  |  |  |  |  |  |
| *PD* | 0.14 | 0.12 | 0.14 | 0.42 | 0.25 | 1.00 |  |  |  |  |  |  |  |  |  |  |  |  |
| *PPF* | 0.35 | 0.22 | 0.32 | 0.04 | -0.13 | 0.49 | 1.00 |  |  |  |  |  |  |  |  |  |  |  |
| *TD* | 0.51 | 0.61 | 0.00 | -0.03 | *-0.72 | -0.59 | -0.27 | 1.00 |  |  |  |  |  |  |  |  |  |  |
| *TFF* | -0.40 | -0.44 | 0.05 | -0.18 | 0.36 | 0.05 | -0.26 | -0.51 | 1.00 |  |  |  |  |  |  |  |  |  |
| *TFF (I)* | *-0.64 | -0.47 | -0.21 | 0.22 | 0.56 | 0.43 | -0.04 | -0.57 | 0.57 | 1.00 |  |  |  |  |  |  |  |  |
| *TFF (C)* | -0.47 | -0.47 | -0.06 | -0.27 | 0.25 | -0.03 | -0.13 | -0.41 | *0.88 | *0.67 | 1.00 |  |  |  |  |  |  |  |
| *TFF (F)* | *-0.83 | *-0.80 | -0.30 | -0.15 | *0.69 | 0.10 | -0.37 | *-0.68 | *0.80 | *0.78 | *0.76 | 1.00 |  |  |  |  |  |  |
| *TFM (C-I)* | 0.09 | -0.19 | 0.00 | *-0.84 | -0.25 | *-0.71 | -0.14 | 0.28 | 0.06 | -0.56 | 0.12 | -0.11 | 1.00 |  |  |  |  |  |
| *TFM (F-C)* | 0.08 | 0.11 | -0.07 | 0.20 | -0.32 | -0.16 | -0.38 | 0.06 | 0.43 | -0.07 | 0.30 | 0.11 | 0.03 | 1.00 |  |  |  |  |
| *TFM (F-I)* | -0.36 | -0.58 | -0.24 | *-0.69 | 0.13 | *-0.66 | -0.32 | -0.07 | 0.14 | -0.38 | 0.10 | 0.22 | *0.81 | 0.10 | 1.00 |  |  |  |
| *PPI* | 0.14 | -0.26 | -0.16 | -0.56 | -0.10 | 0.28 | 0.43 | -0.38 | 0.22 | -0.08 | 0.21 | 0.06 | 0.38 | 0.01 | 0.24 | 1.00 |  |  |
| *PTI* | 0.12 | -0.19 | -0.22 | *-0.65 | -0.33 | -0.07 | 0.04 | -0.07 | 0.42 | -0.06 | 0.49 | 0.14 | 0.57 | 0.36 | 0.32 | *0.81 | 1.00 |  |
| *TPI* | -0.37 | -0.61 | 0.04 | -0.59 | 0.44 | -0.10 | -0.13 | -0.56 | 0.49 | 0.00 | 0.32 | 0.53 | 0.47 | -0.06 | *0.70 | 0.32 | 0.23 | 1.00 |
| *TTI* | -0.20 | -0.48 | 0.04 | -0.58 | 0.44 | 0.36 | 0.33 | -0.61 | 0.36 | 0.29 | 0.33 | 0.42 | 0.21 | -0.51 | 0.19 | *0.64 | 0.36 | *0.65 |

*BD* bout duration; *NP* number of pulses; *NTS* number of tonal segments; *PD* pulse duration; *PPF* pulse peak frequency; *PPI* pulse-pulse interval; *PR* pulse rate; *PTI* pulse-tonal interval; *TD* tonal duration; *TFM* tonal frequency modulation (*I* initial portion of the tonal segment; *C* central portion of the tonal segment; *F* final portion of the tonal segment); *TFF* tonal fundamental frequency; *TPI* tonal-pulse interval; *TR* tonal rate; *TTI* tonal-tonal interval. The mean individual value for each call property was calculated from the means of each bout. All data were log-transformed prior to the analysis. For the descriptions of acoustical properties, see **Table S1**.
