# Supplementary material for: Acoustic Communication at the Water's Edge: Evolutionary Insights from a Mudskipper
Source: PLoS One. 2011 Jun 28;6(6):e21434. doi: 10.1371/journal.pone.0021434 (PMC3125184; doi:10.1371/journal.pone.0021434)
Supplement: Table S4 — Mean acoustic properties of bouts per individual. (DOCX) [file pone.0021434.s011.docx]

**Table S4**. Mean acoustic properties of bouts per individual.

| *#Ind.* | *BD* | *NP* | *NTS* | *PR* | *TR* | *PD* | *PPF* | *TD* | *TFF* | *TFF (I)* | *TFF (C)* | *TFF (F)* | *TFM (C-I)* | *TFM (F-C)* | *TFM (F-I)* | *PPI* | *PTI* | *TPI* | *TTI* |
| --- | --- | --- | --- | --- | --- | --- | --- | --- | --- | --- | --- | --- | --- | --- | --- | --- | --- | --- | --- |
| 1 | 0.461 | 0.602 | 0.301 | 0.413 | 0.184 | 0.024 | 1.725 | 0.157 | 2.275 | 2.371 | 2.386 | 2.384 | 0.978 | 0.391 | 0.906 | 0.081 | 0.000 | 0.319 |  |
| 1 | 0.648 | 1.041 | 0.477 | 0.591 | 0.199 | 0.022 | 1.619 | 0.116 | 2.243 | 2.302 | 2.377 | 2.318 | 1.592 | 1.500 | 1.037 | 0.110 | 0.000 | 0.061 | 0.233 |
| 1 | 0.304 | 0.477 | 0.477 | 0.473 | 0.473 | 0.012 | 1.817 | 0.128 | 2.162 | 2.285 | 2.307 | 2.325 | 1.040 | 0.986 | 1.294 |  | 0.000 | 0.103 | 0.112 |
| 1 | 0.409 | 0.301 | 0.301 | 0.215 | 0.215 | 0.032 | 1.732 | 0.261 | 2.257 | 2.078 | 2.091 | 2.376 | 0.666 | 2.062 | 2.076 |  |  | 0.221 |  |
| 1 | 0.407 | 0.477 | 0.301 | 0.360 | 0.216 | 0.021 | 1.625 | 0.269 | 2.260 | 2.098 | 2.086 | 2.386 | 0.641 | 2.088 | 2.076 |  | 0.000 | 0.216 |  |
| 1 | 0.302 | 0.602 | 0.301 | 0.601 | 0.300 | 0.024 | 1.693 | 0.066 | 2.268 | 2.337 | 2.379 | 2.402 | 1.359 | 1.161 | 1.561 | 0.053 | 0.000 | 0.187 |  |
| 1 | 0.298 | 0.602 | 0.301 | 0.606 | 0.304 | 0.023 | 1.705 | 0.066 | 2.266 | 2.337 | 2.408 | 2.400 | 1.597 | 0.762 | 1.541 | 0.047 | 0.000 | 0.189 |  |
| 1 | 0.171 | 0.301 | 0.301 | 0.487 | 0.487 | 0.030 | 1.916 | 0.150 | 2.197 | 2.344 | 2.316 | 2.351 | 1.164 | 1.262 | 0.671 |  | 0.000 |  |  |
| 1 | 0.128 | 0.301 | 0.301 | 0.592 | 0.592 | 0.021 | 1.680 | 0.113 | 2.257 | 2.335 | 2.376 | 2.402 | 1.355 | 1.201 | 1.574 |  | 0.000 |  |  |
| 1 | 0.098 | 0.301 | 0.301 | 0.693 | 0.693 | 0.017 | 1.797 | 0.084 | 2.227 | 2.332 | 2.356 |  | 1.117 |  |  |  | 0.000 |  |  |
| 2 | 0.507 | 0.699 | 0.477 | 0.448 | 0.279 | 0.018 | 1.904 | 0.136 | 2.230 | 2.168 | 2.373 | 2.332 | 1.953 | 1.350 | 1.835 | 0.021 | 0.163 | 0.071 | 0.158 |
| 2 | 1.003 | 0.903 | 0.301 | 0.249 | 0.045 | 0.019 | 1.715 | 0.185 | 2.228 | 2.046 | 2.337 | 2.335 | 2.029 | 0.335 | 2.025 | 0.415 | 0.059 | 0.025 |  |
| 2 | 0.410 | 0.301 | 0.301 | 0.214 | 0.214 | 0.018 | 1.667 | 0.242 | 2.252 | 2.386 | 2.380 | 2.050 | 0.678 | 2.109 | 2.122 |  |  | 0.250 |  |
| 2 | 1.000 | 1.079 | 0.699 | 0.347 | 0.160 | 0.015 | 1.795 | 0.170 | 2.233 | 2.109 | 2.301 | 2.215 | 1.860 | 1.567 | 1.563 | 0.139 | 0.003 | 0.322 | 0.402 |
| 2 | 0.635 | 0.778 | 0.477 | 0.399 | 0.205 | 0.013 | 1.844 | 0.203 | 2.247 | 1.995 | 2.263 | 2.229 | 1.932 | 1.171 | 1.855 | 0.156 | 0.001 | 0.190 | 0.035 |
| 2 | 0.849 | 1.041 | 0.699 | 0.423 | 0.220 | 0.013 | 1.759 | 0.139 | 2.229 | 2.318 | 2.356 | 2.343 | 1.305 | 1.021 | 1.128 | 0.112 | 0.009 | 0.225 | 0.275 |
| 3 | 0.189 | 0.301 | 0.301 | 0.453 | 0.453 | 0.010 | 1.803 | 0.182 | 2.151 | 2.203 | 2.264 | 2.246 | 1.396 | 0.920 | 1.244 |  | 0.000 |  |  |
| 3 | 0.683 | 0.845 | 0.477 | 0.410 | 0.183 | 0.015 | 1.707 | 0.216 | 2.152 | 2.221 | 2.177 | 2.218 | 1.764 | 1.252 | 1.875 | 0.108 | 0.000 | 0.205 | 0.392 |
| 3 | 0.590 | 0.778 | 0.477 | 0.436 | 0.228 | 0.017 | 1.805 | 0.145 | 2.207 | 2.288 | 2.337 | 2.329 | 1.383 | 0.669 | 1.317 | 0.204 | 0.000 | 0.131 | 0.256 |
| 3 | 0.116 | 0.000 | 0.301 | 0.000 | 0.631 |  |  | 0.116 | 2.174 | 2.275 | 2.411 | 2.428 | 1.848 | 1.046 | 1.906 |  |  |  |  |
| 4 | 0.708 | 0.778 | 0.301 | 0.346 | 0.095 | 0.020 | 1.749 | 0.179 | 2.202 | 2.271 | 2.328 | 2.168 | 1.430 | 1.823 | 1.609 | 0.322 | 0.000 | 0.033 |  |
| 4 | 0.348 | 0.602 | 0.301 | 0.536 | 0.258 | 0.017 | 1.726 | 0.100 | 2.201 | 2.248 | 2.299 | 2.325 | 1.358 | 1.131 | 1.548 | 0.154 | 0.001 |  |  |
| 4 | 0.549 | 0.602 | 0.301 | 0.339 | 0.144 | 0.031 | 1.674 | 0.117 | 2.207 | 2.207 | 2.325 | 2.190 | 1.712 | 1.761 | 0.855 | 0.360 | 0.025 | 0.220 |  |
| 4 | 0.225 | 0.477 | 0.301 | 0.596 | 0.393 | 0.031 | 1.708 | 0.111 | 2.155 | 2.188 | 2.272 | 2.297 | 1.529 | 1.079 | 1.651 | 0.100 | 0.000 |  |  |
| 4 | 0.632 | 0.602 | 0.301 | 0.281 | 0.115 | 0.039 | 1.523 | 0.100 | 2.202 | 2.290 | 2.330 | 2.280 | 1.292 | 1.384 | 0.748 | 0.376 | 0.000 |  |  |
| 4 | 0.810 | 1.079 | 0.699 | 0.479 | 0.239 | 0.026 | 1.721 | 0.116 | 2.182 | 2.279 | 2.313 | 2.288 | 1.281 | 1.147 | 1.432 | 0.135 | 0.018 | 0.113 | 0.216 |
| 4 | 0.812 | 1.079 | 0.699 | 0.478 | 0.238 | 0.016 | 1.751 | 0.112 | 2.189 | 2.289 | 2.314 | 2.316 | 1.126 | 0.905 | 1.137 | 0.166 | 0.018 | 0.122 | 0.219 |
| 4 | 0.299 | 0.477 | 0.301 | 0.480 | 0.303 | 0.023 | 1.552 | 0.109 | 2.180 | 2.284 | 2.267 | 2.289 | 0.933 | 1.033 | 0.508 | 0.108 | 0.117 |  |  |
| 4 | 1.025 | 0.903 | 0.477 | 0.238 | 0.082 | 0.023 | 1.741 | 0.202 | 2.153 | 2.245 | 2.285 | 2.267 | 1.258 | 0.952 | 1.006 | 0.461 | 0.003 | 0.093 | 0.185 |
| 4 | 1.021 | 0.699 | 0.301 |  |  | 0.033 | 1.648 | 0.262 | 2.186 | 2.247 | 2.316 | 2.284 | 1.502 | 1.199 | 1.229 | 0.569 | 0.092 |  |  |
| 4 | 0.913 | 1.146 | 0.477 | 0.449 | 0.107 | 0.043 | 1.928 | 0.223 | 2.141 | 2.261 | 2.303 | 2.262 | 1.285 | 1.274 | 0.883 | 0.179 | 0.000 | 0.081 | 0.204 |
| 4 | 1.222 | 1.415 | 0.602 | 0.414 | 0.076 | 0.059 | 1.821 | 0.186 | 2.207 | 2.264 | 2.325 | 2.289 | 1.463 | 1.256 | 1.218 | 0.169 | 0.004 | 0.069 | 0.565 |
| 4 | 0.377 | 0.477 | 0.301 | 0.389 | 0.237 | 0.033 | 1.813 | 0.167 | 2.209 | 2.286 | 2.321 | 2.301 | 1.231 | 1.014 | 0.886 |  | 0.017 | 0.233 |  |
| 4 | 0.734 | 0.954 | 0.301 | 0.449 | 0.089 | 0.044 | 1.941 | 0.158 | 2.198 | 2.308 | 2.314 | 2.235 | 0.614 | 1.546 | 1.506 | 0.145 | 0.000 | 0.243 |  |
| 4 | 0.803 | 0.845 | 0.301 | 0.326 | 0.074 | 0.038 | 1.898 | 0.143 | 2.198 | 2.234 | 2.290 | 2.207 | 1.397 | 1.546 | 1.050 | 0.299 | 0.000 | 0.161 |  |
| 4 | 0.314 | 0.602 | 0.301 | 0.583 | 0.289 | 0.025 | 1.742 | 0.114 | 2.246 | 2.323 | 2.381 | 2.349 | 1.493 | 1.262 | 1.140 | 0.064 | 0.000 | 0.152 |  |
| 4 | 0.581 | 0.778 | 0.301 | 0.444 | 0.132 | 0.033 | 1.962 | 0.137 | 2.250 | 2.354 | 2.373 | 2.243 | 1.064 | 1.795 | 1.714 | 0.179 | 0.000 | 0.190 |  |
| 4 | 0.468 | 0.301 | 0.301 | 0.181 | 0.181 | 0.018 | 1.888 | 0.228 | 2.276 | 2.299 | 2.366 | 2.371 | 1.534 | 0.569 | 1.567 |  | 0.343 |  |  |
| 4 | 0.708 | 0.954 | 0.301 | 0.470 | 0.095 | 0.027 | 1.751 | 0.137 | 2.222 | 2.305 | 2.363 | 2.316 | 1.475 | 1.393 | 0.791 | 0.143 | 0.001 | 0.272 |  |
| 5 | 0.179 | 0.477 | 0.301 | 0.692 | 0.471 | 0.021 | 1.820 | 0.079 | 2.209 | 2.274 | 2.361 | 2.368 | 1.630 | 0.698 | 1.669 | 0.083 | 0.000 |  |  |
| 5 | 1.160 | 1.362 | 0.778 | 0.421 | 0.137 | 0.041 | 1.827 | 0.176 | 2.166 | 2.146 | 2.131 | 2.143 | 1.171 | 0.715 | 1.059 | 0.166 | 0.000 | 0.117 | 0.322 |
| 5 | 1.040 | 1.079 | 0.477 | 0.323 | 0.079 | 0.024 | 1.794 | 0.129 | 2.171 | 2.267 | 2.260 | 2.324 | 1.266 | 1.709 | 1.528 | 0.282 | 0.008 | 0.149 | 0.377 |
| 5 | 0.992 | 0.903 | 0.301 | 0.254 | 0.047 | 0.049 | 1.715 | 0.145 | 2.198 | 2.301 | 2.299 | 2.272 | 0.298 | 1.113 | 1.145 | 0.338 |  | 0.174 |  |
| 5 | 0.204 | 0.477 | 0.301 | 0.637 | 0.426 | 0.021 | 1.763 | 0.121 | 2.246 | 2.323 | 2.371 | 2.337 | 1.406 | 1.272 | 0.892 | 0.072 | 0.000 |  |  |
| 5 | 0.155 | 0.301 | 0.301 | 0.522 | 0.522 | 0.025 | 1.620 | 0.127 | 2.250 | 2.346 | 2.368 | 2.366 | 1.099 | 0.370 | 1.050 |  |  | 0.013 |  |
| 5 | 0.583 | 0.778 | 0.301 | 0.442 | 0.131 | 0.039 | 1.732 | 0.281 | 2.163 | 2.316 | 2.274 | 2.332 | 1.307 | 1.447 | 0.940 | 0.093 |  | 0.173 |  |
| 6 | 0.083 | 0.301 | 0.301 | 0.761 | 0.761 | 0.021 | 1.982 | 0.068 | 2.342 | 2.442 | 2.454 | 2.436 | 0.946 | 1.100 | 0.677 |  | 0.000 |  |  |
| 6 | 0.604 | 0.954 | 0.602 | 0.562 | 0.300 | 0.030 | 1.780 | 0.146 | 2.260 | 2.367 | 2.394 | 2.355 | 1.202 | 1.351 | 1.330 | 0.056 | 0.001 | 0.090 | 0.160 |
| 6 | 0.832 | 1.079 | 0.602 | 0.462 | 0.181 | 0.036 | 1.790 | 0.118 | 2.209 | 2.303 | 2.309 | 2.316 | 1.126 | 1.195 | 0.897 | 0.166 | 0.000 | 0.085 | 0.459 |
| 6 | 0.666 | 0.903 | 0.602 | 0.467 | 0.262 | 0.037 | 1.742 | 0.188 | 2.235 | 2.321 | 2.354 | 2.297 | 1.254 | 1.462 | 1.080 | 0.077 | 0.002 | 0.111 | 0.134 |
| 7 | 0.809 | 0.602 | 0.477 | 0.191 | 0.136 | 0.019 | 1.654 | 0.134 | 2.284 | 2.370 | 2.415 | 2.419 | 1.428 | 0.929 | 1.462 | 0.567 | 0.061 | 0.413 | 0.730 |
| 7 | 0.124 | 0.000 | 0.301 | 0.000 | 0.604 |  |  | 0.124 | 2.293 | 2.270 | 2.386 | 2.433 | 1.765 | 1.460 | 1.935 |  |  |  |  |
| 7 | 0.118 | 0.000 | 0.301 | 0.000 | 0.624 |  |  | 0.118 | 2.293 | 2.268 | 2.408 | 2.439 | 1.854 | 1.305 | 1.957 |  |  |  |  |
| 7 | 0.172 | 0.301 | 0.301 | 0.486 | 0.486 | 0.016 | 1.898 | 0.108 | 2.306 | 2.349 | 2.461 | 2.442 | 1.823 | 1.111 | 1.738 |  | 0.067 |  |  |
| 7 | 0.230 | 0.301 | 0.301 | 0.385 | 0.385 | 0.026 | 1.679 | 0.078 | 2.293 | 2.342 | 2.381 | 2.430 | 1.341 | 1.476 | 1.707 |  | 0.159 |  |  |
| 7 | 0.455 | 0.602 | 0.477 | 0.418 | 0.318 | 0.065 | 1.802 | 0.062 | 2.243 | 2.362 | 2.356 | 2.386 | 1.000 | 1.232 | 1.151 | 0.247 | 0.060 | 0.000 | 0.029 |
| 7 | 0.140 | 0.000 | 0.301 | 0.000 | 0.560 |  |  | 0.140 | 2.293 | 2.379 | 2.436 | 2.428 | 1.544 | 0.809 | 1.471 |  |  |  |  |
| 7 | 0.259 | 0.477 | 0.301 | 0.538 | 0.347 | 0.010 | 1.896 | 0.084 | 2.284 | 2.354 | 2.400 | 2.297 | 1.425 | 1.735 | 1.457 | 0.194 | 0.000 |  |  |
| 8 | 0.584 | 0.845 | 0.477 | 0.494 | 0.232 | 0.020 | 1.693 | 0.165 | 2.194 | 2.293 | 2.312 | 2.298 | 0.999 | 0.878 | 0.633 | 0.110 | 0.045 | 0.106 | 0.134 |
| 8 | 0.894 | 0.954 | 0.477 | 0.336 | 0.111 | 0.014 | 1.832 | 0.132 | 2.174 | 2.298 | 2.304 | 2.267 | 0.597 | 1.372 | 1.345 | 0.323 | 0.018 | 0.052 | 0.345 |
| 8 | 0.587 | 0.954 | 0.477 | 0.579 | 0.230 | 0.041 | 1.854 | 0.141 | 2.183 | 2.235 | 2.270 | 2.266 | 1.177 | 0.992 | 1.208 | 0.091 | 0.006 | 0.023 | 0.332 |
| 8 | 0.524 | 0.845 | 0.301 | 0.552 | 0.155 | 0.030 | 1.945 | 0.072 | 2.196 | 2.303 | 2.354 | 2.305 | 1.410 | 1.393 | 0.302 | 0.134 | 0.000 |  |  |
| 8 | 0.507 | 0.699 | 0.477 | 0.449 | 0.280 | 0.030 | 1.691 | 0.169 | 2.186 | 2.313 | 2.325 | 2.274 | 0.896 | 1.388 | 1.351 | 0.143 | 0.011 | 0.068 | 0.134 |
| 8 | 0.539 | 0.602 | 0.301 | 0.346 | 0.148 | 0.036 | 1.918 | 0.179 | 2.198 | 2.284 | 2.345 | 2.363 | 1.474 | 1.031 | 1.597 | 0.256 | 0.033 |  |  |
| 8 | 0.823 | 0.845 | 0.301 | 0.314 | 0.071 | 0.018 | 1.736 | 0.325 | 2.182 | 2.321 | 2.308 | 2.311 | 0.864 | 0.398 | 0.764 | 0.308 | 0.000 | 0.059 |  |
| 8 | 0.669 | 0.602 | 0.301 | 0.260 | 0.105 | 0.029 | 1.878 | 0.132 | 2.126 | 2.286 | 2.361 | 2.174 | 1.571 | 1.911 | 1.655 | 0.407 | 0.000 |  |  |
| 8 | 0.352 | 0.477 | 0.477 | 0.415 | 0.415 | 0.018 | 1.856 | 0.129 | 2.179 | 2.296 | 2.300 | 2.202 | 1.650 | 1.619 | 1.597 |  | 0.004 | 0.091 | 0.128 |
| 8 | 0.261 | 0.477 | 0.301 | 0.535 | 0.345 | 0.033 | 1.977 | 0.124 | 2.222 | 2.178 | 2.222 | 2.205 | 1.226 | 0.861 | 1.023 | 0.125 | 0.000 |  |  |
| 8 | 0.184 | 0.477 | 0.000 | 0.681 | 0.000 | 0.037 | 1.979 |  |  |  |  |  |  |  |  | 0.130 |  |  |  |
| 8 | 0.318 | 0.699 | 0.301 | 0.673 | 0.285 | 0.028 | 1.959 | 0.158 | 2.228 | 2.252 | 2.358 | 2.351 | 1.703 | 0.682 | 1.669 | 0.069 | 0.000 | 0.014 |  |
| 8 | 0.228 | 0.477 | 0.301 | 0.591 | 0.389 | 0.030 | 1.715 | 0.122 | 2.247 | 2.363 | 2.366 | 2.376 | 0.400 | 0.799 | 0.892 | 0.088 | 0.000 |  |  |
| 9 | 0.688 | 1.041 | 0.477 | 0.554 | 0.181 | 0.019 | 1.723 | 0.096 | 2.220 | 2.291 | 2.336 | 2.353 | 1.506 | 1.053 | 1.489 | 0.097 | 0.008 | 0.196 | 0.306 |
| 9 | 0.305 | 0.301 | 0.301 | 0.297 | 0.297 | 0.029 | 1.851 | 0.231 | 2.269 | 2.368 | 2.386 | 2.339 | 1.036 | 1.416 | 1.209 |  |  | 0.096 |  |
| 9 | 0.314 | 0.301 | 0.301 | 0.288 | 0.288 | 0.027 | 1.803 | 0.250 | 2.268 | 2.368 | 2.397 | 2.356 | 1.228 | 1.370 | 0.879 |  |  | 0.086 |  |
| 10 | 0.730 | 1.114 | 0.602 | 0.574 | 0.227 | 0.013 | 1.751 | 0.156 | 2.220 | 2.252 | 2.332 | 2.290 | 1.574 | 1.491 | 1.589 | 0.086 | 0.081 | 0.044 | 0.192 |
| 10 | 0.719 | 0.903 | 0.301 | 0.423 | 0.092 | 0.017 | 1.705 | 0.315 | 2.208 | 2.188 | 2.344 | 2.207 | 1.829 | 1.784 | 0.889 | 0.167 |  | 0.029 |  |
| 10 | 0.628 | 0.602 | 0.000 | 0.285 | 0.000 | 0.014 | 1.681 |  |  |  |  |  |  |  |  | 0.410 |  |  |  |
| 10 | 0.834 | 0.699 | 0.301 | 0.227 | 0.069 | 0.027 | 1.709 | 0.215 | 2.210 | 2.290 | 2.330 | 2.312 | 1.292 | 0.986 | 1.038 | 0.523 | 0.000 | 0.101 |  |

#Ind. = ID number of each individual; number of bouts = 78. Other abbreviations as in **Tab. S3**. These means were utilised to conduct the MANOVA and the 19 ANOVAs, after logarithmic transformation (see main text, material and methods).
