# Supplementary material for: Acoustic Communication at the Water's Edge: Evolutionary Insights from a Mudskipper
Source: PLoS One. 2011 Jun 28;6(6):e21434. doi: 10.1371/journal.pone.0021434 (PMC3125184; doi:10.1371/journal.pone.0021434)
Supplement: Table S5 — Individual means. (DOCX) [file pone.0021434.s012.docx]

**Table S5**. Individual means.

| *#Ind.* | *BD* | *NP* | *NTS* | *PR* | *TR* | *PD* | *PPF* | *TD* | *TFF* | *TFF (I)* | *TFF (C)* | *TFF (F)* | *TFM (C-I)* | *TFM (F-C)* | *TFM (F-I)* | *PPI* | *PTI* | *TPI* | *TTI* |
| --- | --- | --- | --- | --- | --- | --- | --- | --- | --- | --- | --- | --- | --- | --- | --- | --- | --- | --- | --- |
| 1 | 1.3 | 2.7 | 1.2 | 2.3 | 1.5 | 0.1 | 53.9 | 0.4 | 173.8 | 194.9 | 208.6 | 234.9 | 17.1 | 36.3 | 42.3 | 0.2 | 0.0 | 0.6 | 0.5 |
| 2 | 5.2 | 6.3 | 2.3 | 1.3 | 0.6 | 0.0 | 60.4 | 0.5 | 171.5 | 155.2 | 216.2 | 181.8 | 62.3 | 34.9 | 70.4 | 0.6 | 0.1 | 0.6 | 0.7 |
| 3 | 1.9 | 3.0 | 1.5 | 1.3 | 1.6 | 0.0 | 58.4 | 0.5 | 147.4 | 176.1 | 201.2 | 204.7 | 43.4 | 9.5 | 47.5 | 0.4 | 0.0 | 0.5 | 1.1 |
| 4 | 4.6 | 6.4 | 1.5 | 1.7 | 0.5 | 0.1 | 59.4 | 0.4 | 158.0 | 187.1 | 208.7 | 189.8 | 22.5 | 23.1 | 19.4 | 0.8 | 0.1 | 0.4 | 1.0 |
| 5 | 5.2 | 7.1 | 1.7 | 2.1 | 1.0 | 0.1 | 56.3 | 0.4 | 158.1 | 192.2 | 199.3 | 204.0 | 18.5 | 16.6 | 18.1 | 0.5 | 0.0 | 0.3 | 1.2 |
| 6 | 3.2 | 6.8 | 2.5 | 2.8 | 1.8 | 0.1 | 67.3 | 0.4 | 182.9 | 228.9 | 239.6 | 225.1 | 13.0 | 18.9 | 10.5 | 0.3 | 0.0 | 0.2 | 0.9 |
| 7 | 1.3 | 1.3 | 1.3 | 1.0 | 1.9 | 0.1 | 61.8 | 0.3 | 192.4 | 216.9 | 254.0 | 257.0 | 38.6 | 21.3 | 47.0 | 1.3 | 0.2 | 0.8 | 2.2 |
| 8 | 2.6 | 4.3 | 1.3 | 2.2 | 0.7 | 0.1 | 71.6 | 0.4 | 155.3 | 192.9 | 208.2 | 192.7 | 20.0 | 19.4 | 20.8 | 0.6 | 0.0 | 0.1 | 0.7 |
| 9 | 2.0 | 4.0 | 1.3 | 1.5 | 0.8 | 0.1 | 61.4 | 0.6 | 178.1 | 219.9 | 235.5 | 222.6 | 19.0 | 19.3 | 17.2 | 0.3 | 0.0 | 0.3 | 1.0 |
| 10 | 4.4 | 6.5 | 1.3 | 1.5 | 0.3 | 0.0 | 50.6 | 0.7 | 162.2 | 175.0 | 215.5 | 186.0 | 40.5 | 32.8 | 18.1 | 1.1 | 0.1 | 0.1 | 0.6 |

Abbreviations as in **Table S4**. Each means was obtained as the mean of the bouts’ means; the individual means were used to calculate the between-individual CV, or *CVb*, and the grand means (see also **Table 2**).
